# Supplementary material for: Influence of femoral anteversion angle and neck-shaft angle on muscle forces and joint loading during walking
Source: PLoS One. 2023 Oct 12;18(10):e0291458. doi: 10.1371/journal.pone.0291458 (PMC10569567; doi:10.1371/journal.pone.0291458)
Supplement: S1 File — (DOCX) [file pone.0291458.s001.docx]

Supplementary material to accompany: Influence of femoral anteversion angle and neck-shaft angle on muscle forces and joint loading during walking

Hans Kainz^1^*, Gabriel T. Mindler^2,3^, Andreas Kranzl^3,4^

^1^ Centre for Sport Science and University Sports, Department of Biomechanics, Kinesiology and Computer Science in Sport, Neuromechanics Research Group, University of Vienna, Vienna, Austria

^2^ Department of Pediatric Orthopaedics, Orthopaedic Hospital Speising, Vienna, Austria

^3^Vienna Bone and Growth Center, Vienna, Austria

^4^Laboratory for Gait and Movement Analysis, Orthopaedic Hospital Speising, Vienna, Austria

# * corresponding author: Ass-Prof. Dr. Hans Kainz, hans.kainz@univie.ac.at

Content of this document: Additional figures and table to support the findings and conclusions of our study

**Table S1.** Detailed results from the multiple regression analysis.

|  | **Overall** | | **AVA** | | **NSA** | |  |
| --- | --- | --- | --- | --- | --- | --- | --- |
|  | **R^2** | **p** | **B** | **p** | **B** | **p** | **constant** |
| **Joint contact forces** |  |  |  |  |  |  |  |
| Hip JCF | 0.86 | <0.001 | 0.026 | <0.001 | 0.017 | <0.001 | 3.495 |
| Knee JCF | 0.6 | <0.001 | 0.019 | <0.001 | 0.019 | <0.001 | 3.148 |
| Ankle JCF | 0.03 | 0.726 |  |  |  |  |  |
| **Muscle moments** |  |  |  |  |  |  |  |
| hip adduction | 0.62 | <0.001 | -6.73E-05 | <0.001 | 6.11E-05 | <0.001 | 0.013 |
| hip abduction | 0.62 | <0.001 | 6.94E-05 | <0.001 | -5.80E-05 | <0.001 | -0.057 |
| hip flexion | 0.836 | <0.001 | 0.001 | <0.001 | 0.0001972 | 0.015 | 0.056 |
| hip extension | 0.837 | <0.001 | -0.000763 | <0.001 | -0.0001975 | 0.014 | -0.041 |
| hip int rotation | 0.69 | <0.001 | 5.12E-05 | 0.091 | -0.0001966 | <0.001 | 0.016 |
| hip ext rotation | 0.686 | <0.001 | -4.416E-05 | 0.143 | 0.00019681 | <0.001 | -0.013 |
| kne extension | 0.566 | <0.001 | 0.00024482 | 0.003 | 0.00029892 | <0.001 | 0.055 |
| knee flexion | 0.566 | <0.001 | -0.0002451 | 0.003 | -0.0002986 | <0.001 | -0.028 |
| **CCI** |  |  |  |  |  |  |  |
| hip flex/ext | 0.922 | <0.001 | -0.0034027 | <0.001 | -0.0002225 | 0.304 | 0.202 |
| hip ab-/adduction | 0.468 | <0.001 | 0.00029963 | 0.028 | -0.0004735 | 0.001 | 0.63 |
| hip int/ext rotation | 0.838 | <0.001 | 0.001 | <0.001 | -0.003 | <0.001 | 0.035 |
| knee flex/erxt | 0.266 | 0.033 | -9.089E-06 | 0.97 | 0.00066707 | 0.01 | -0.315 |
| **Muscle activation** |  |  |  |  |  |  |  |
| hip adduction | 0.712 | <0.001 | 9.9049E-05 | 0.001 | -0.0001723 | <0.001 | 0.035 |
| hip abduction | 0.717 | <0.001 | 0.003 | <0.001 | 0.001 | 0.022 | 0.156 |
| hip flexion | 0.82 | <0.001 | 0.00124922 | <0.001 | 0.00010434 | 0.42 | 0.073 |
| hip extension | 0.62 | <0.001 | 0.00116336 | 0.001 | 0.00046393 | <0.001 | 0.101 |
| hip int rotation | 0.82 | <0.001 | 0.00141289 | <0.001 | 0.00067186 | <0.001 | 0.124 |
| hip ext rotation | 0.56 | <0.001 | 0.00091832 | <0.001 | -5.389E-05 | 0.759 | 0.057 |
| kne extension | 0.67 | <0.001 | 0.00143253 | <0.001 | 0.00039048 | 0.082 | 0.107 |
| knee flexion | 0.54 | <0.001 | 0.000575 | 0.029 | 0.0010928 | <0.001 | 0.087 |


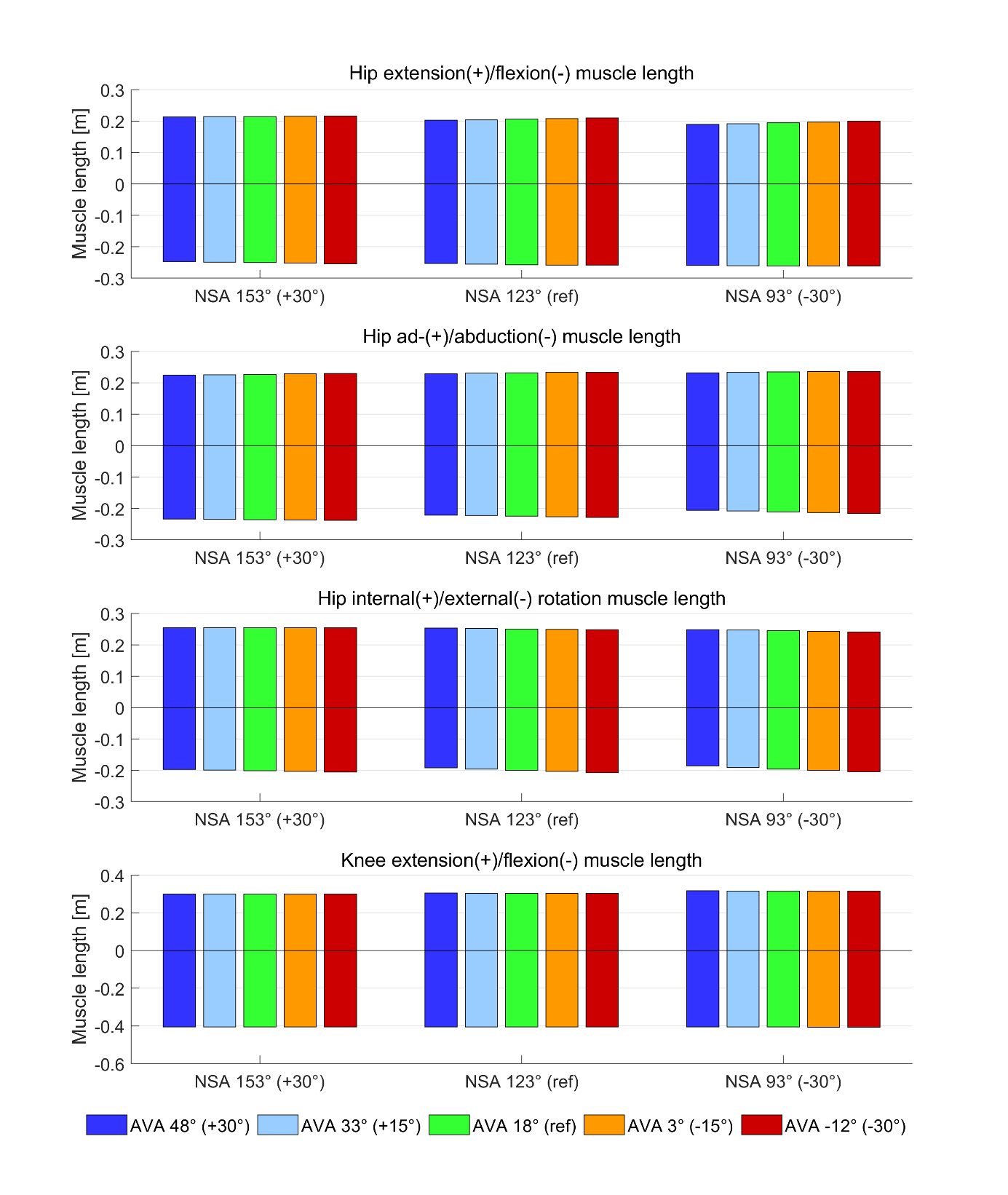


**Figure S1.** Average muscle-tendon length during the stance phase of gait obtained from agonist and antagonist muscle groups.


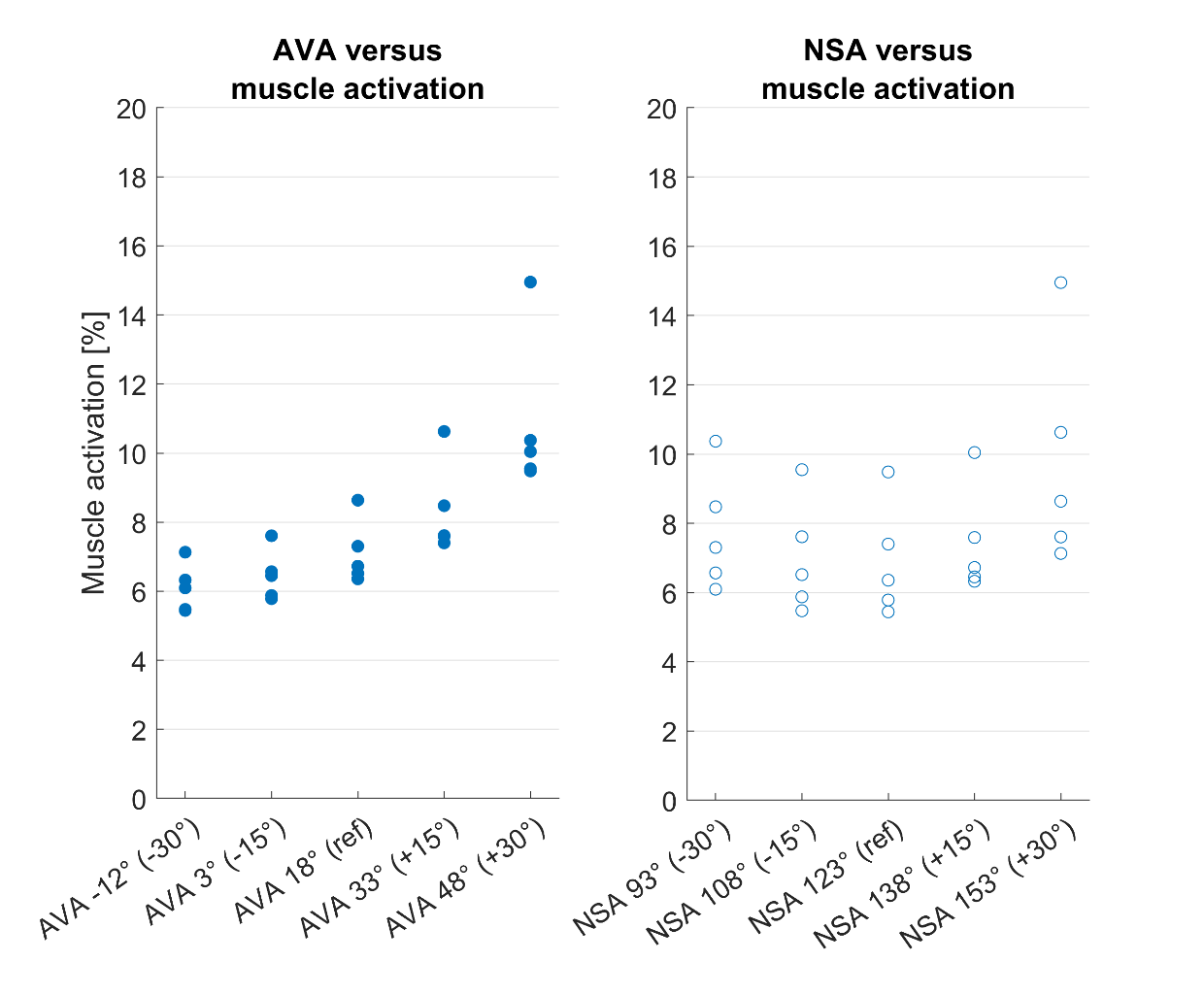


**Figure S2.** Scatterplots showing the relationship between the femoral geometry (AVA and NSA) and average muscle activations during the stance phase of gait. Filled circles indicate significant correlations (p<0.05).


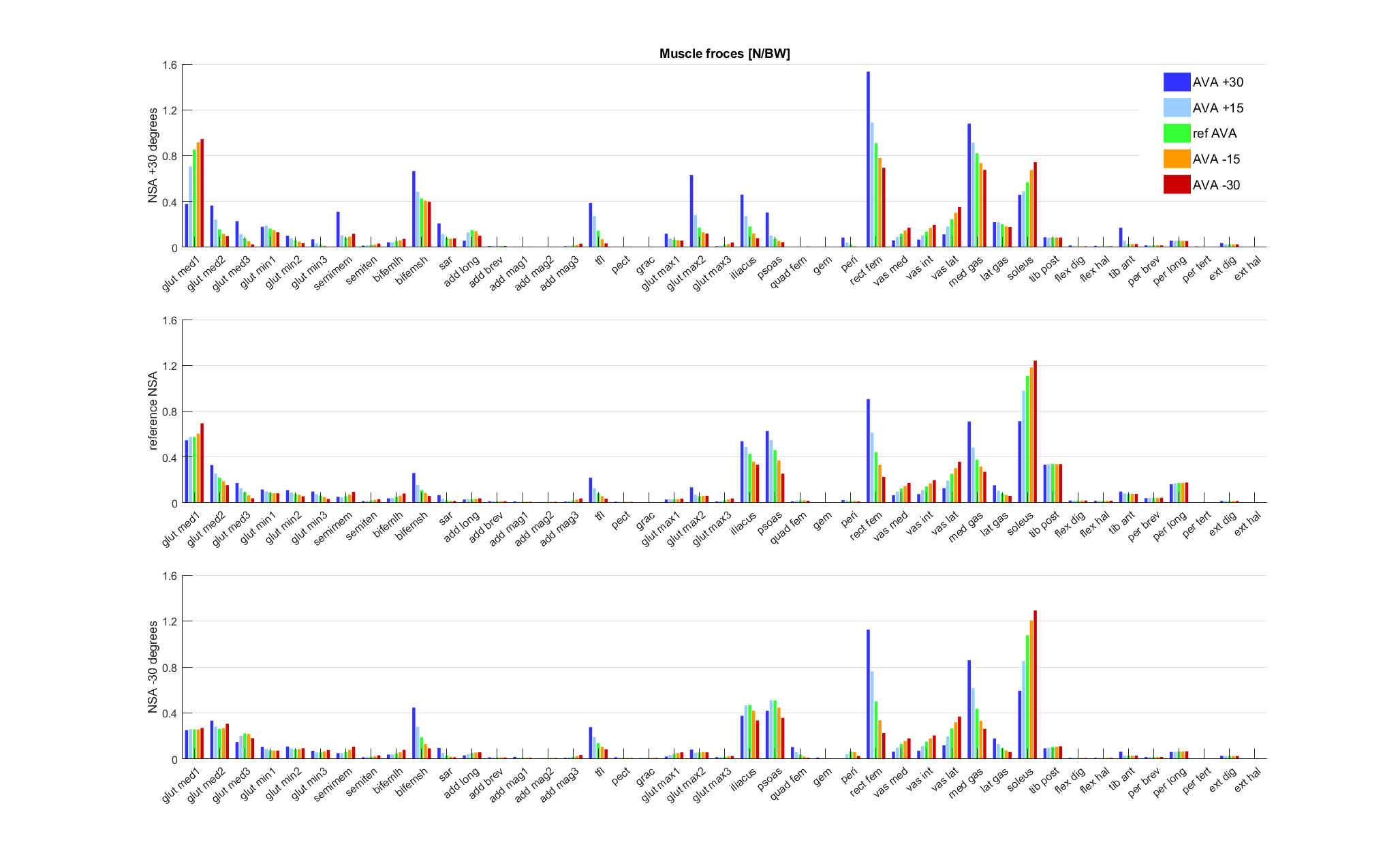


**Figure S3.** Average muscle-tendon forces during the stance phase of gait.


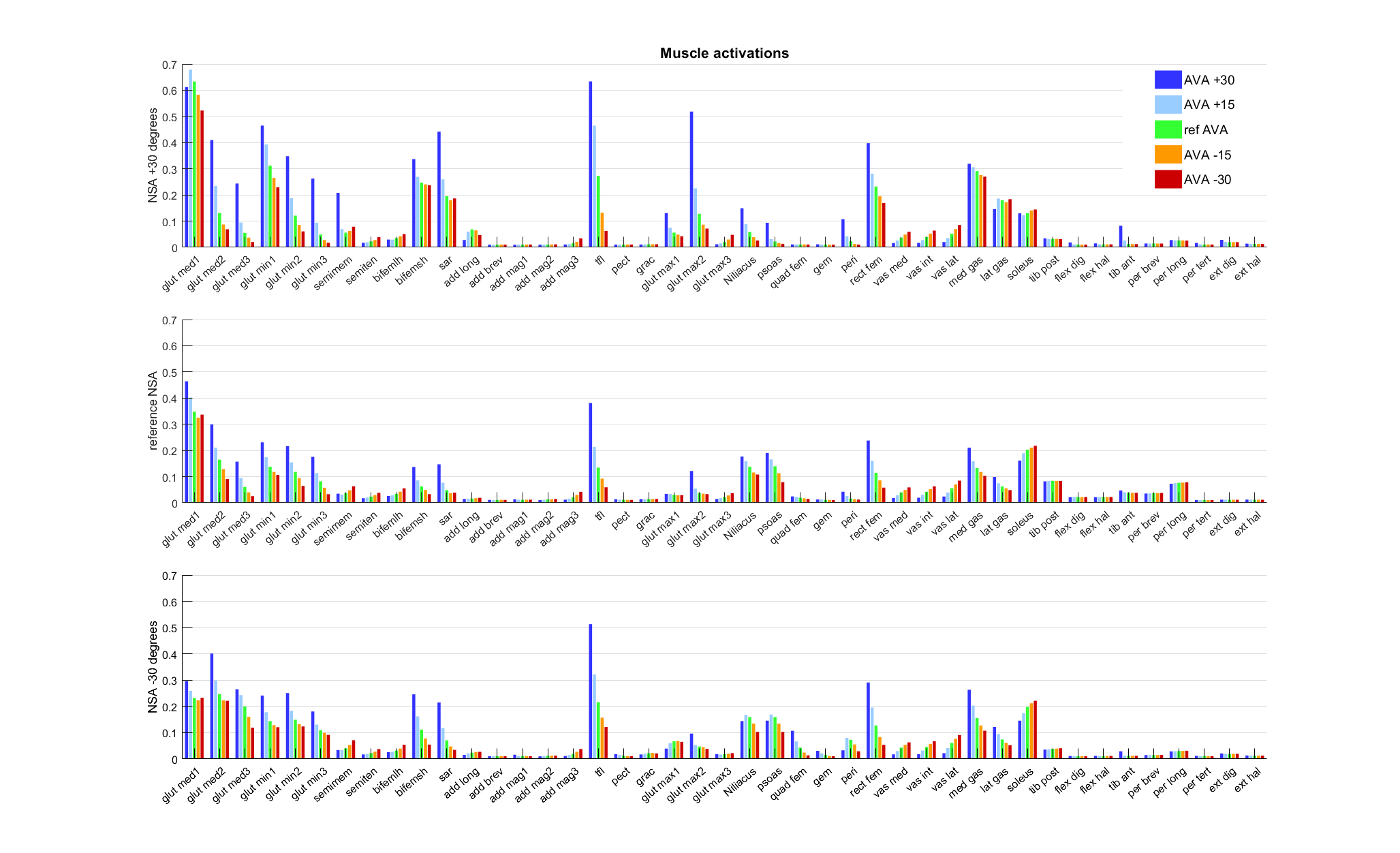


**Figure S4.** Average muscle activation during the stance phase of gait.


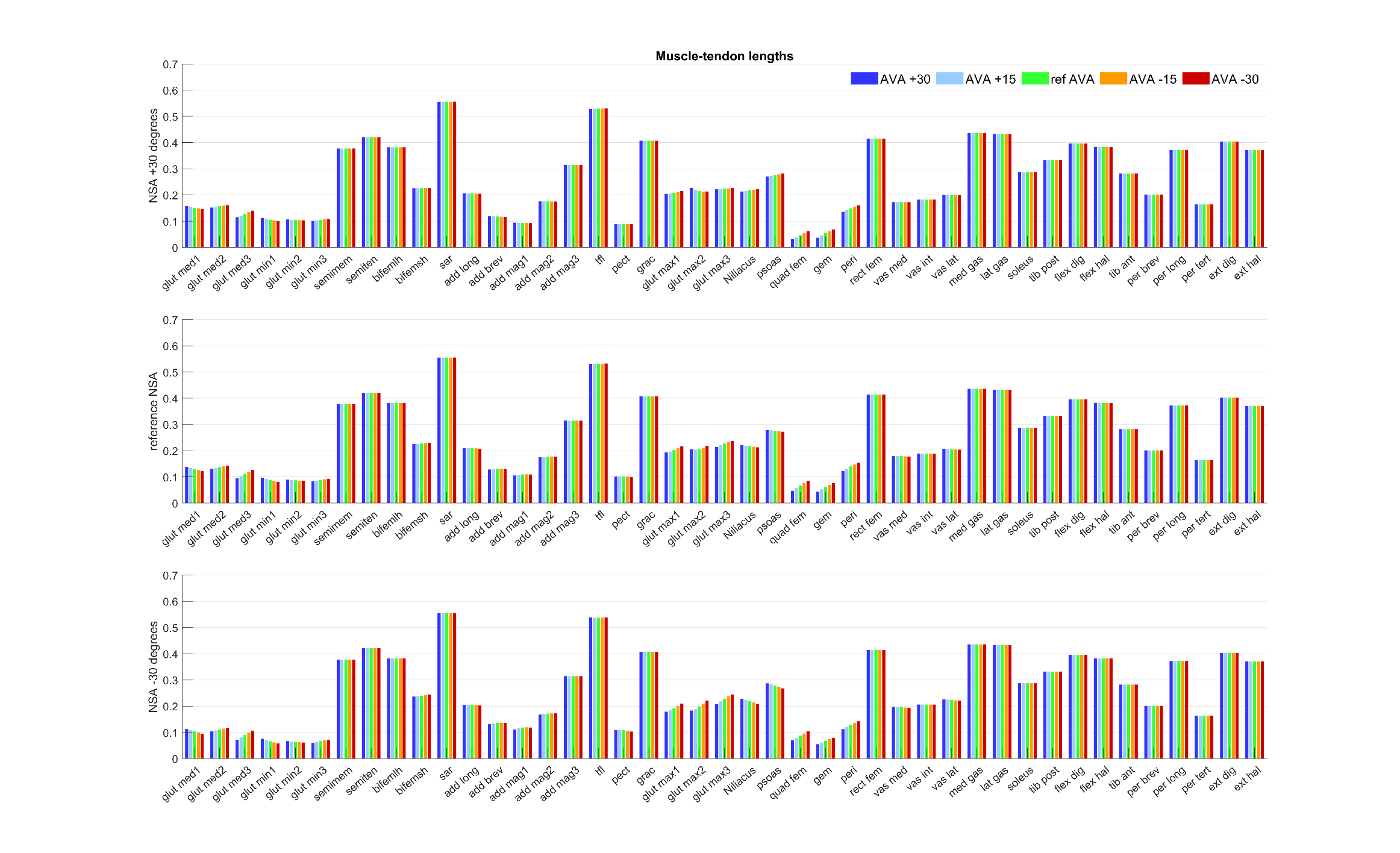


**Figure S5.** Average muscle-tendon length during the stance phase of gait.


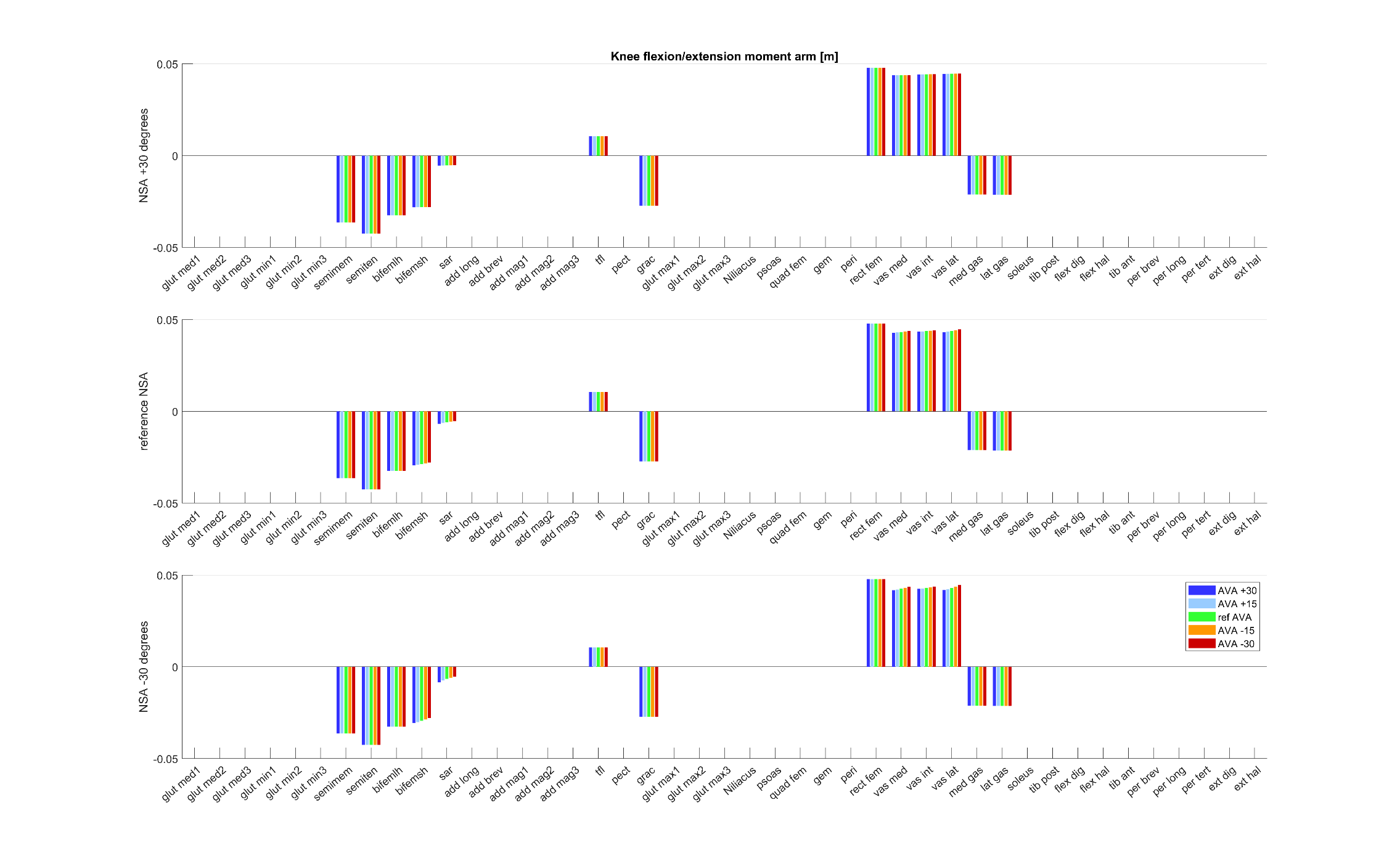


**Figure S6.** Average muscle knee flexion/extension moment arm during the stance phase of gait.


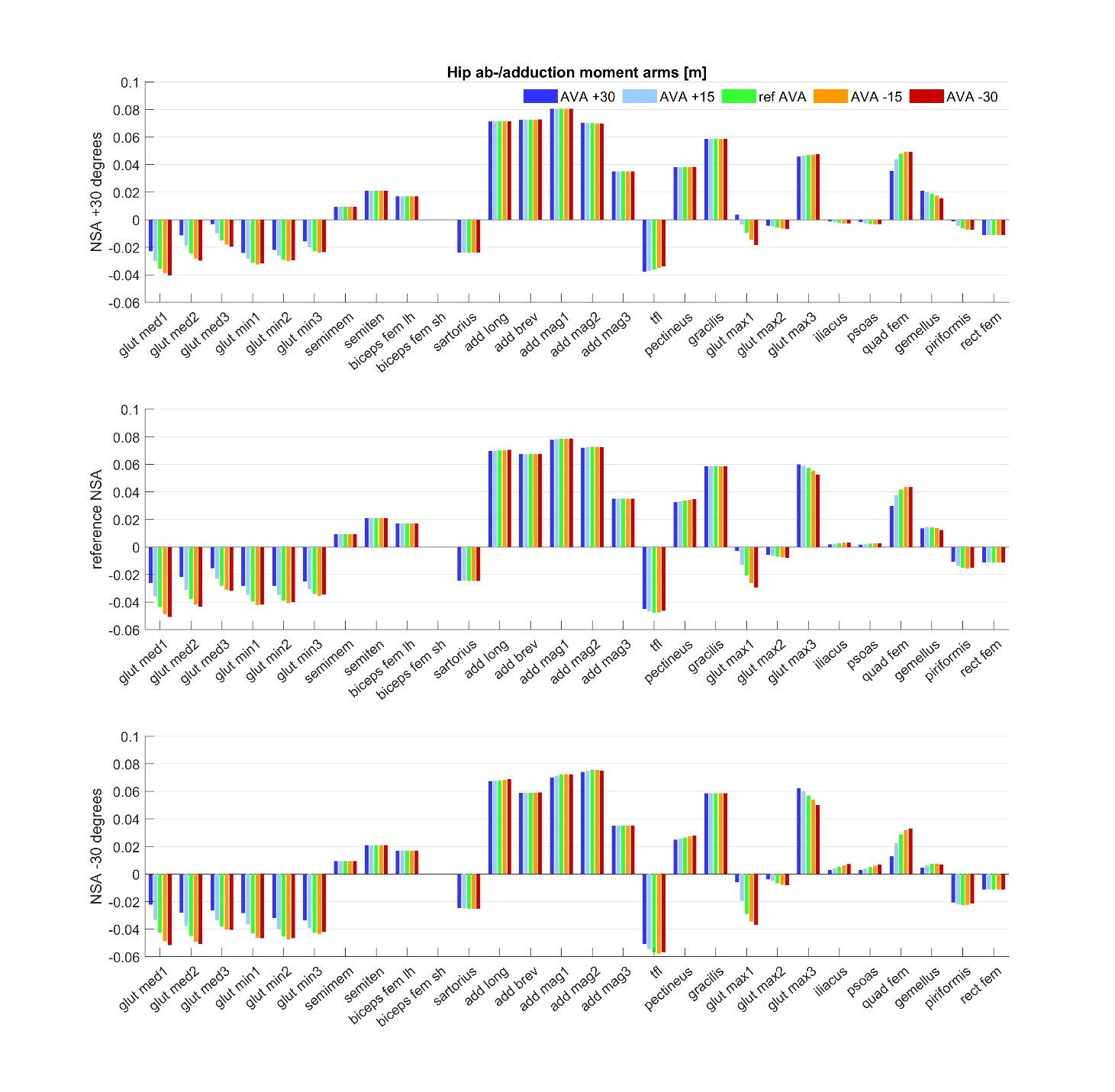


**Figure S7.** Average muscle hip ab-/adduction moment arm during the stance phase of gait.


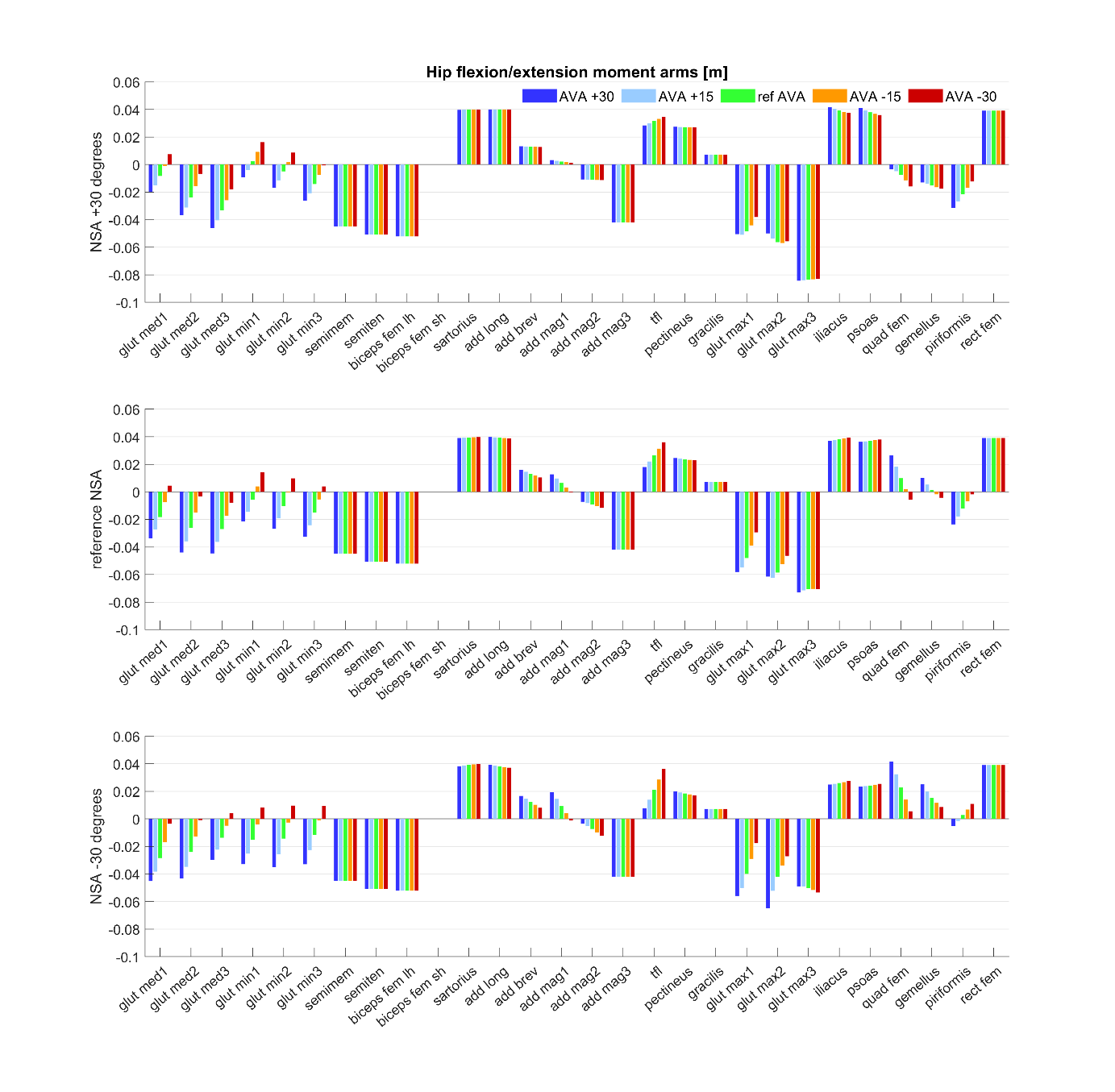

**Figure S8.** Average muscle hip flexion/extension moment arm during the stance phase of gait.


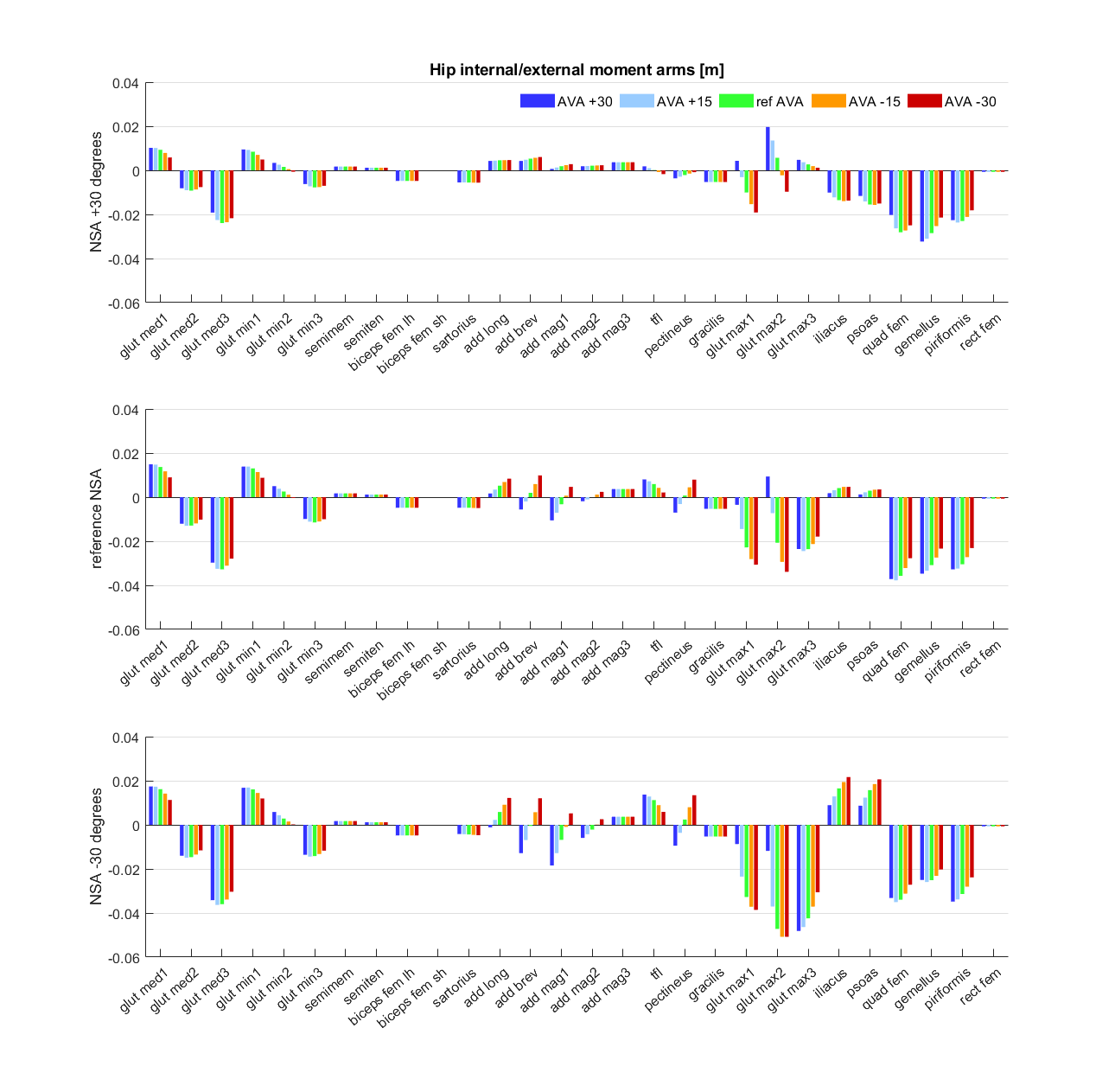


**Figure S9.** Average muscle hip internal/external rotation moment arm during the stance phase of gait.

ADDITIONAL PLOTS FOR ALTERED NECK-SHAFT-ANGLES (NSA)


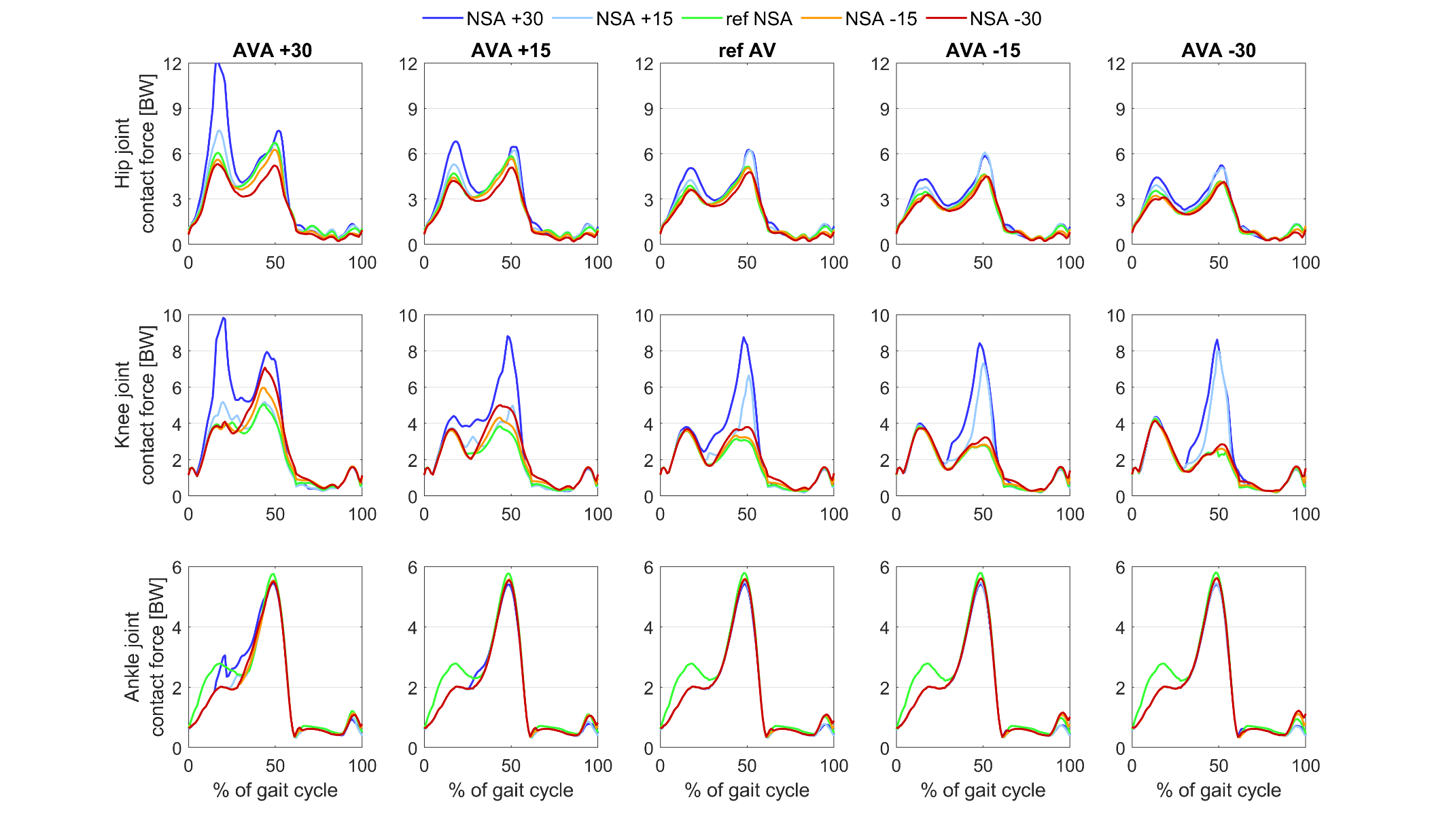


**Figure S10.** Hip, knee and ankle joint contact forces obtained with the different models.


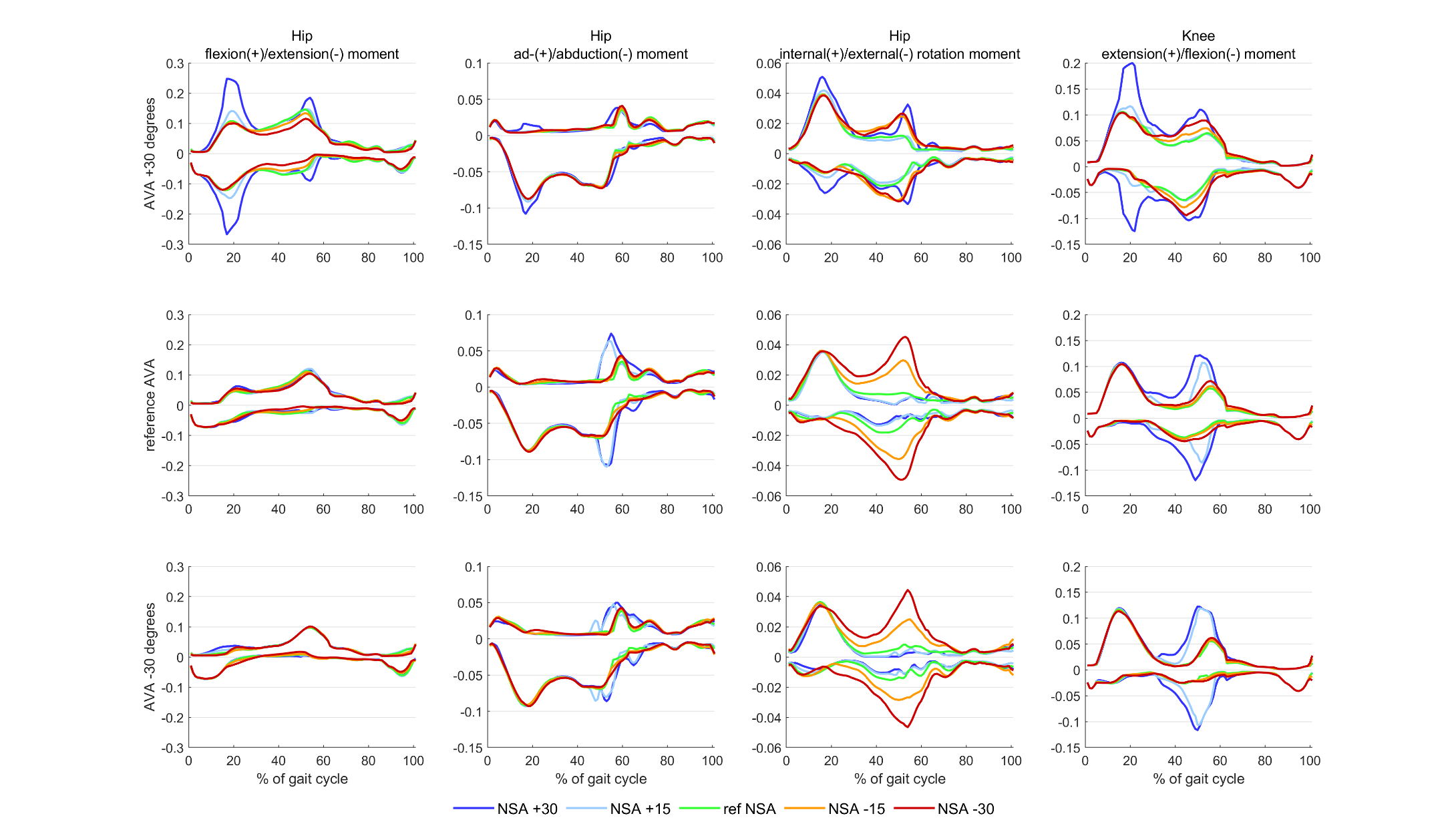


**Figure S11.** Agonist and antagonist muscle moments obtained with model with different femoral geometry. Both, altered AVA and NSA, had an influence on the obtained muscle moments.


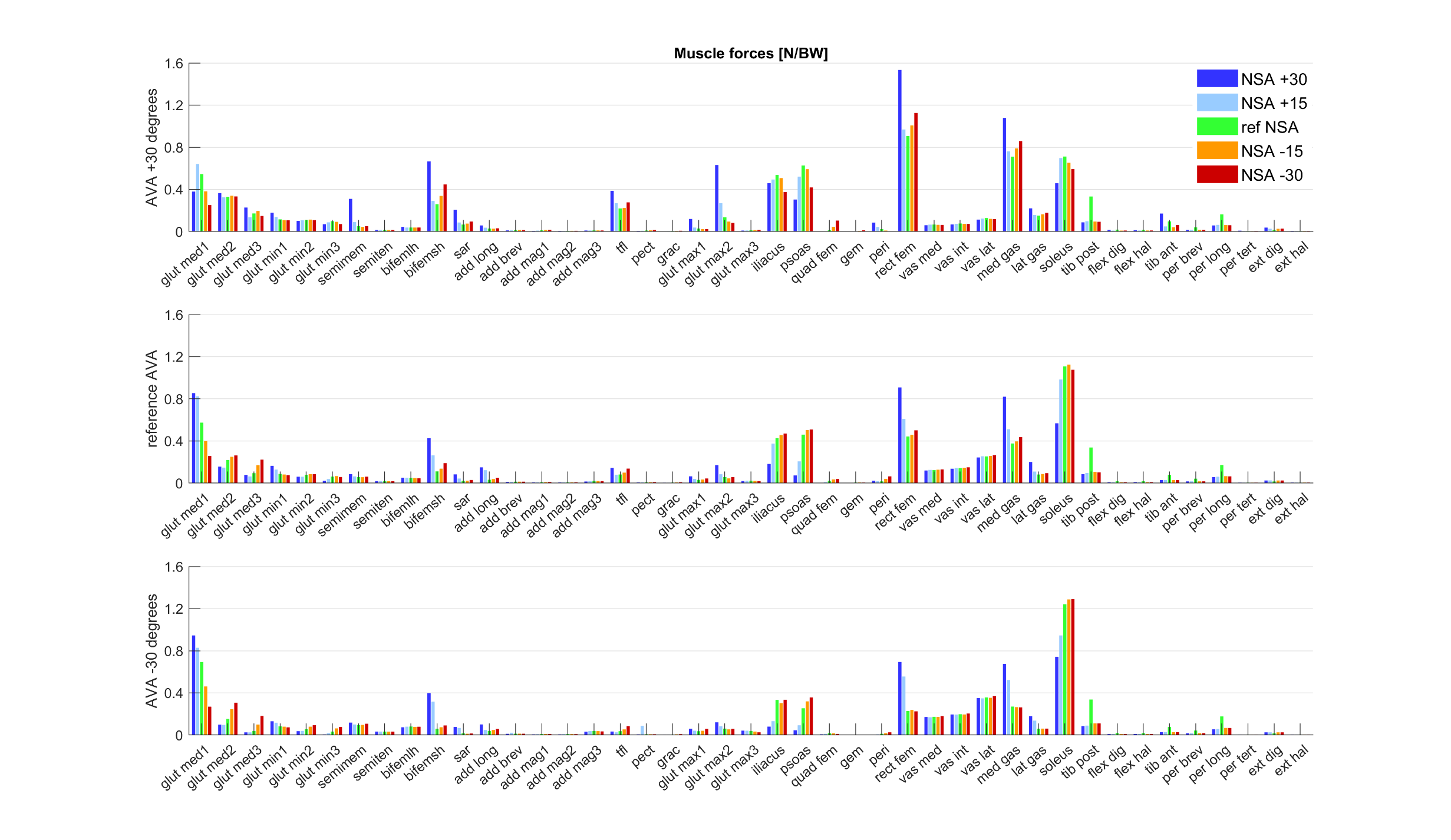


**Figure S12.** Average muscle-tendon forces during the stance phase of gait.


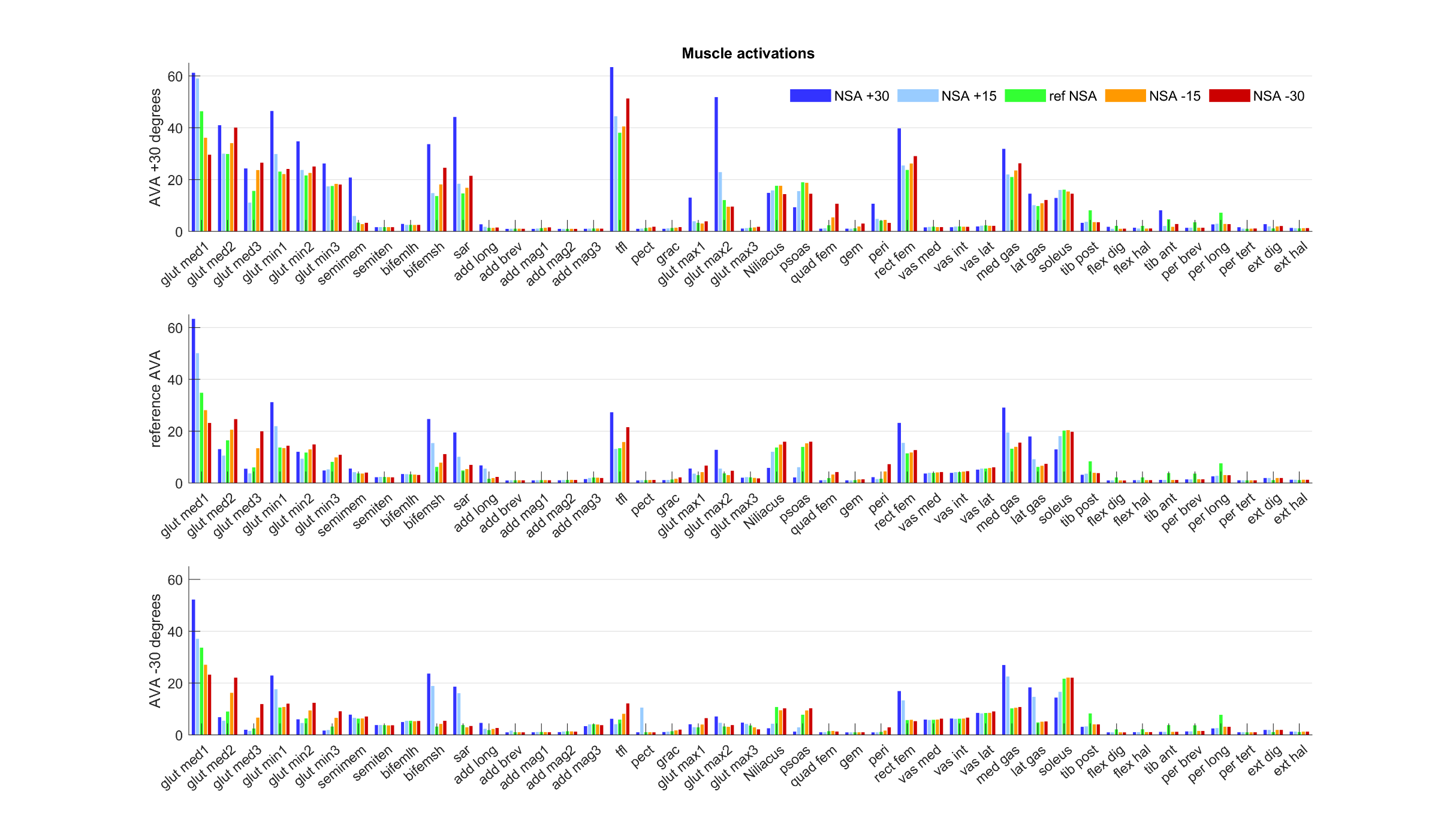


**Figure S13.** Average muscle activations during the stance phase of gait.


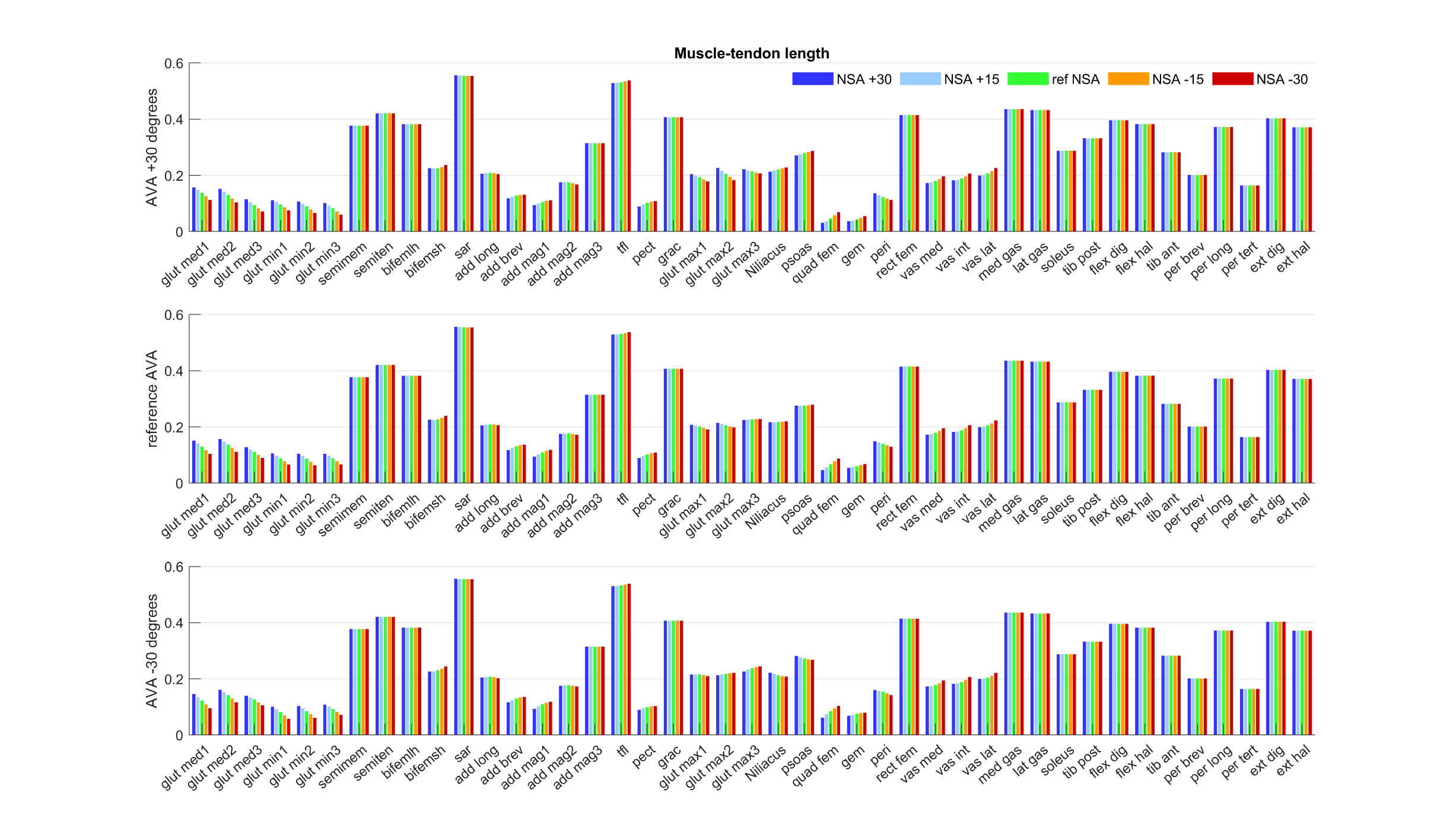


**Figure S14.** Average muscle-tendon lengths during the stance phase of gait.


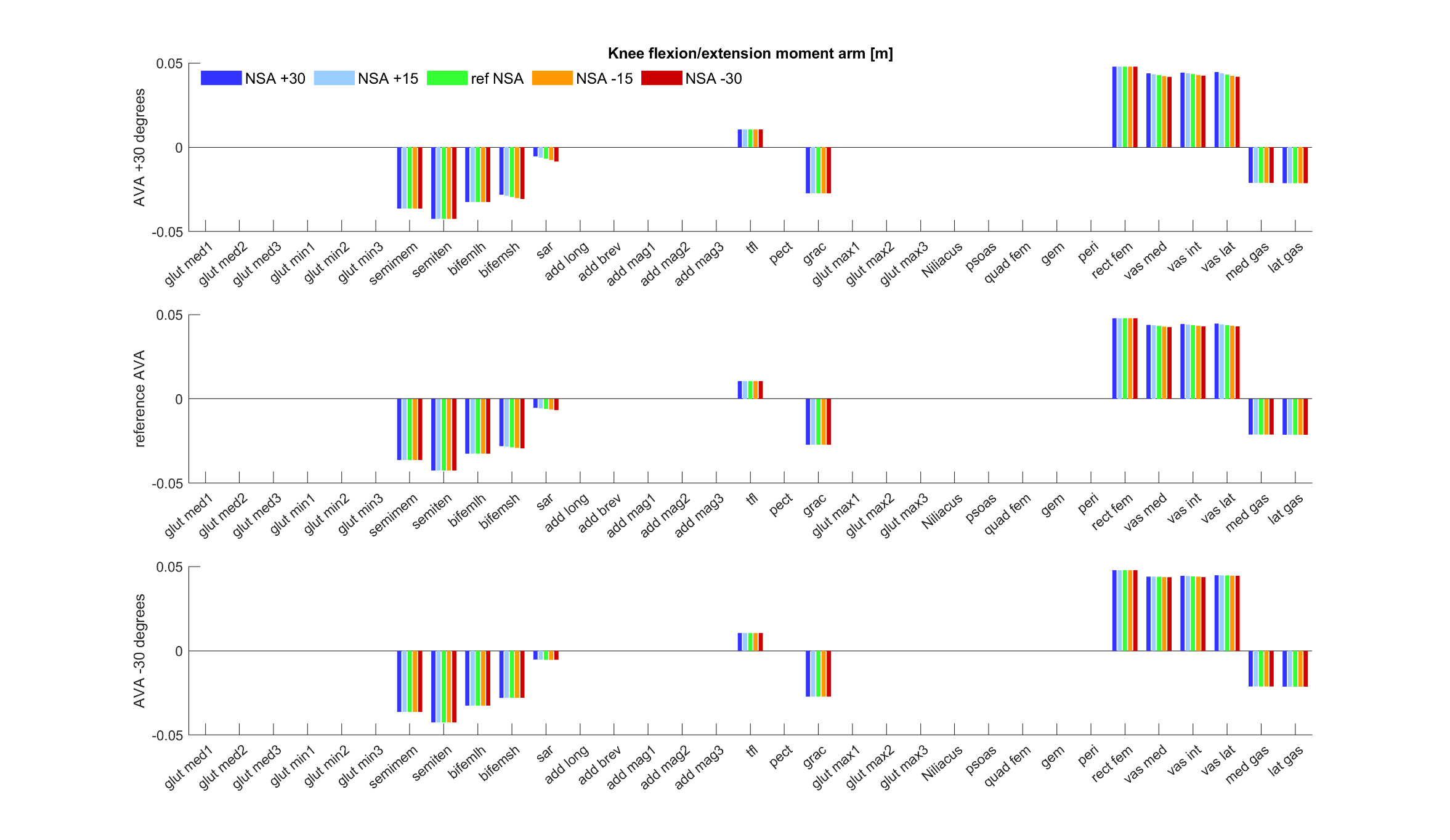


**Figure S15.** Average muscle knee flexion/extension moment arms during the stance phase of gait.


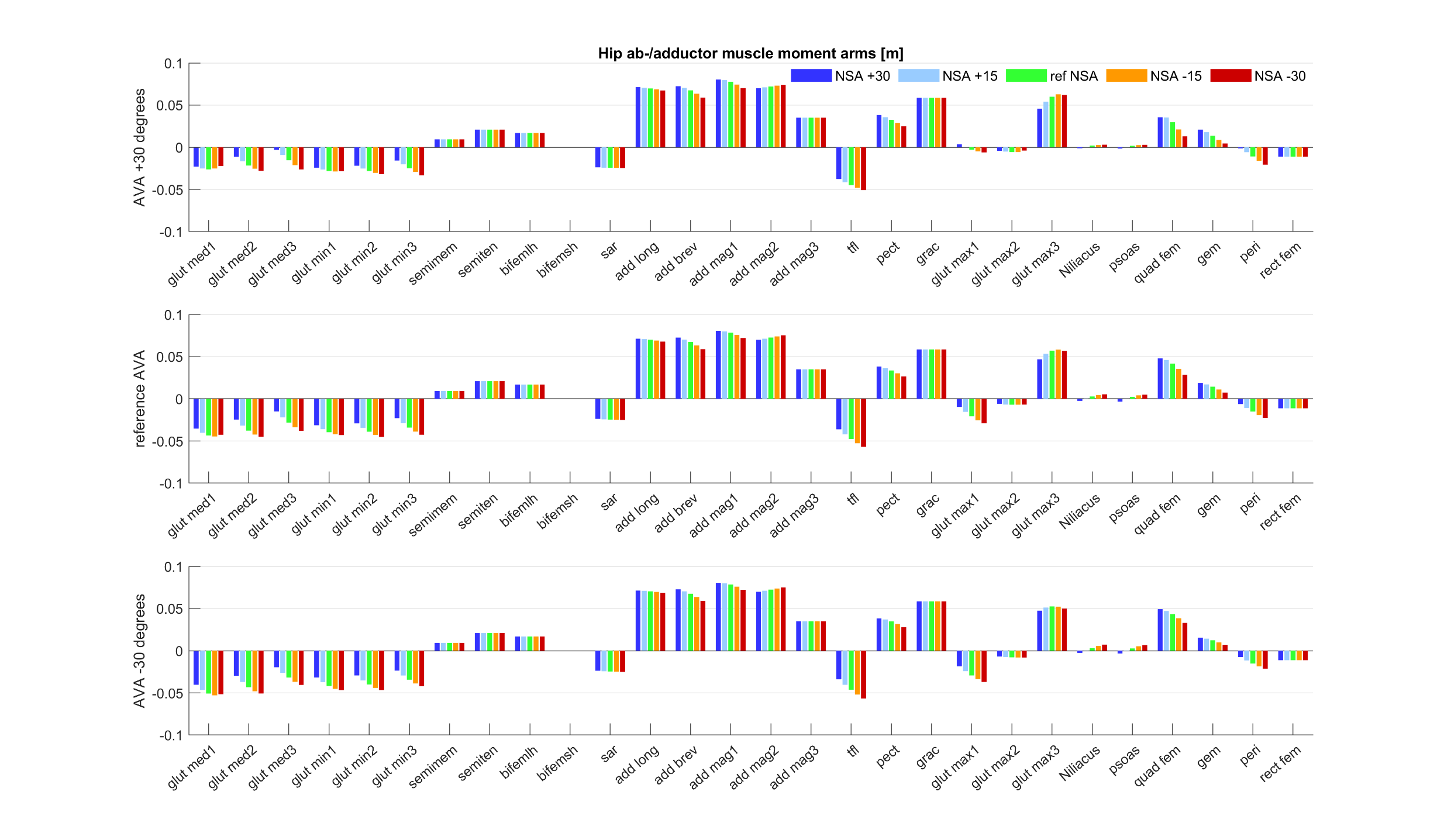


**Figure S16.** Average muscle hip ab-/adduction moment arms during the stance phase of gait.


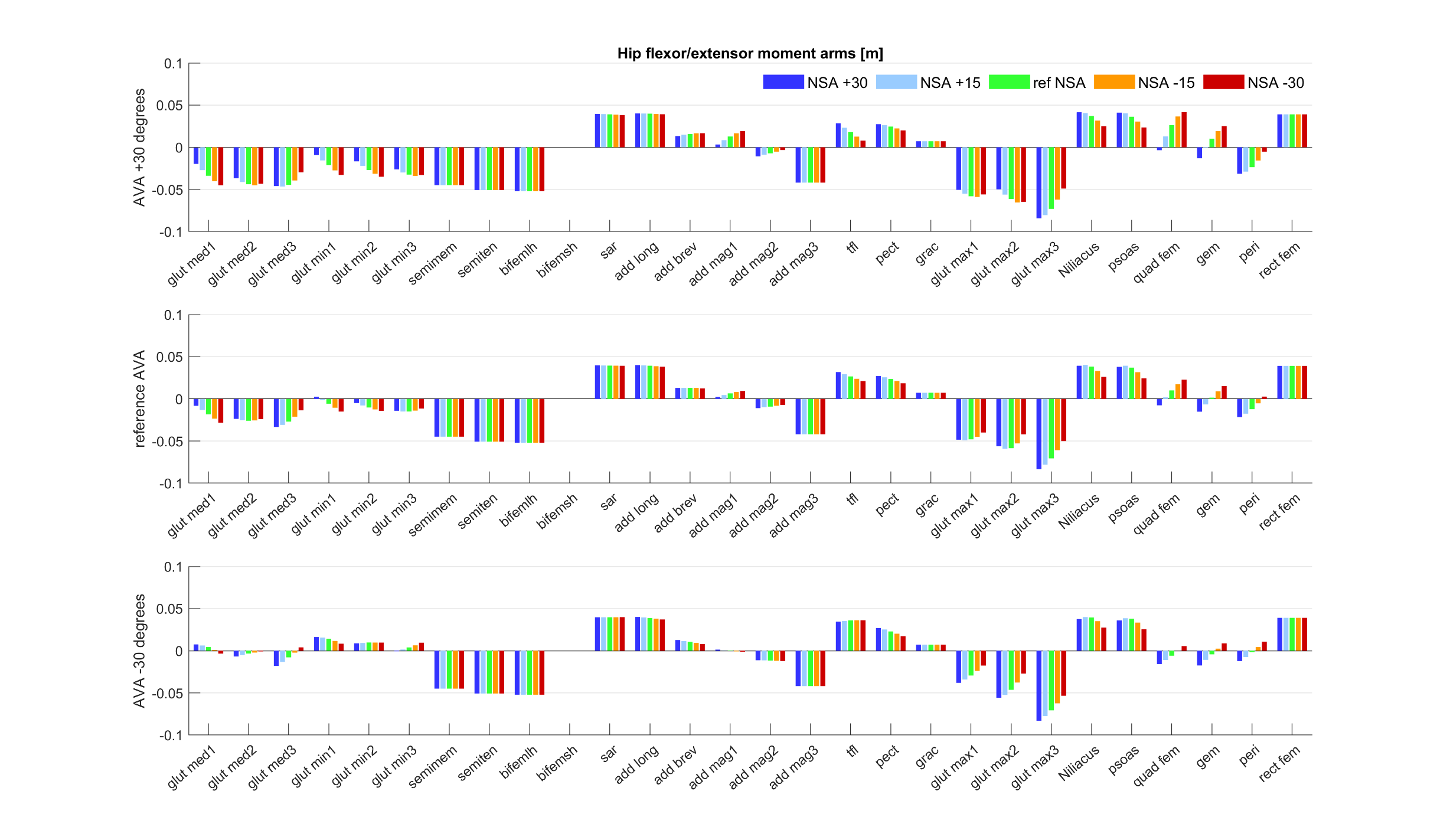


**Figure S17.** Average muscle hip flexion/extension moment arms during the stance phase of gait.


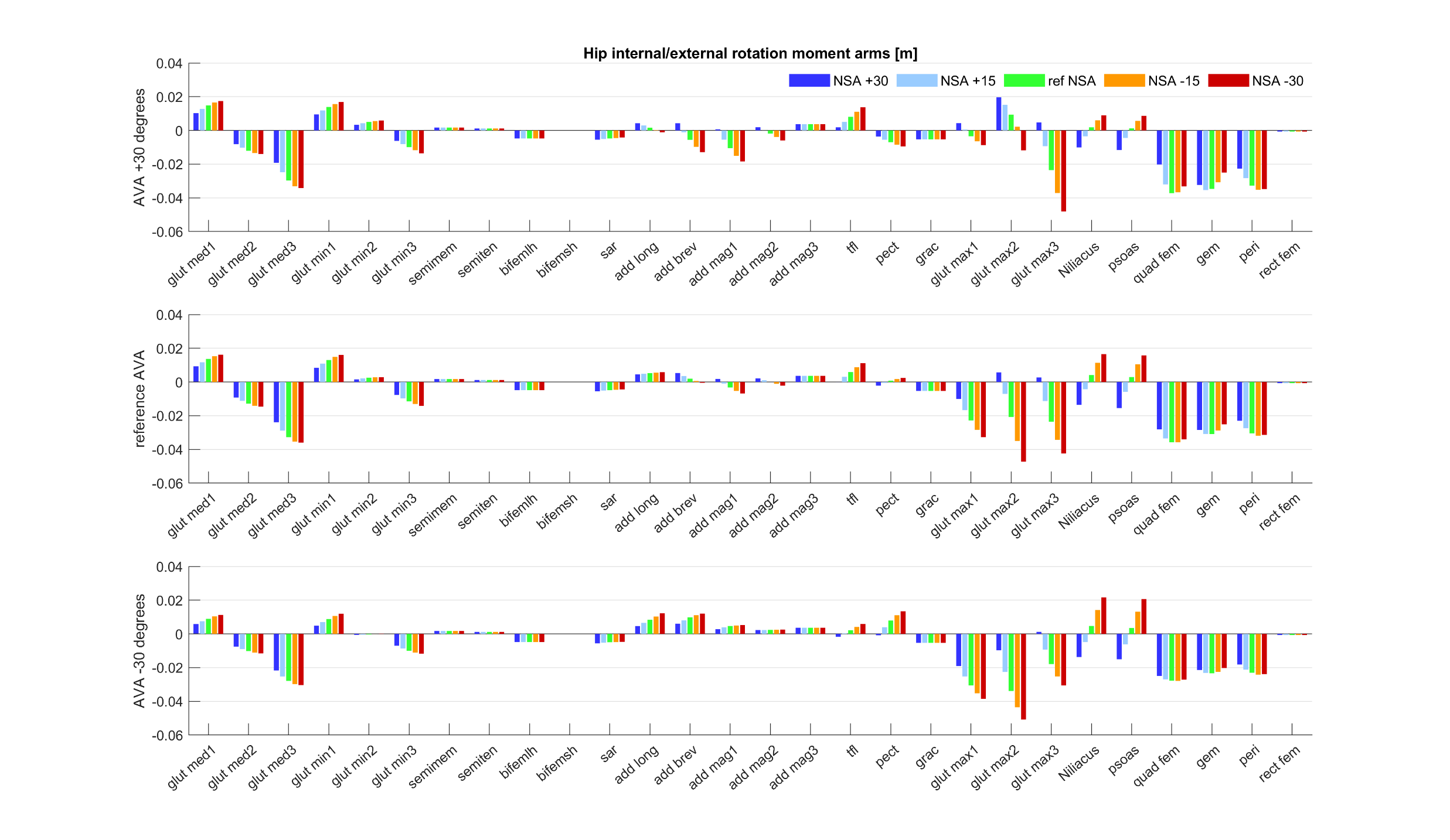


**Figure S18.** Average muscle hip internal/external rotation moment arms during the stance phase of gait.

ADDITIONAL PLOTS SHOWING THE INTERACTION BETWEEN AVA, NSA and JCF, CCI, and muscle moments


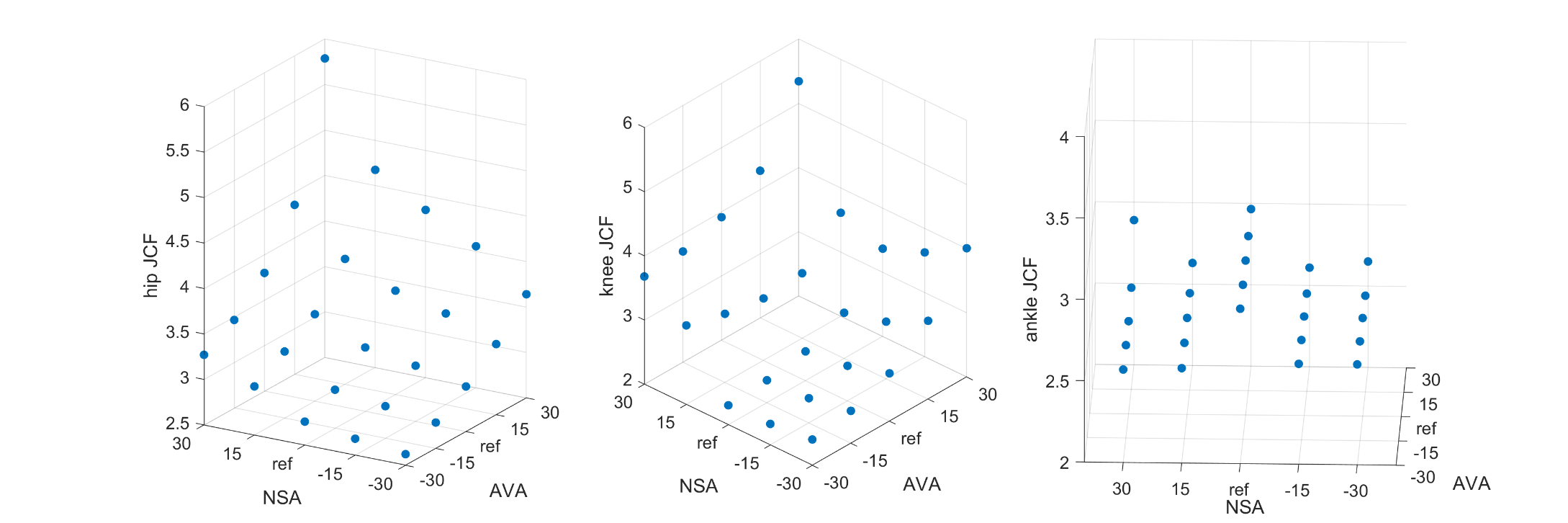


**Figure S19.** Scatterplots showing the relationship between the femoral geometry (AVA and NSA) and joint contact forces (JCF).


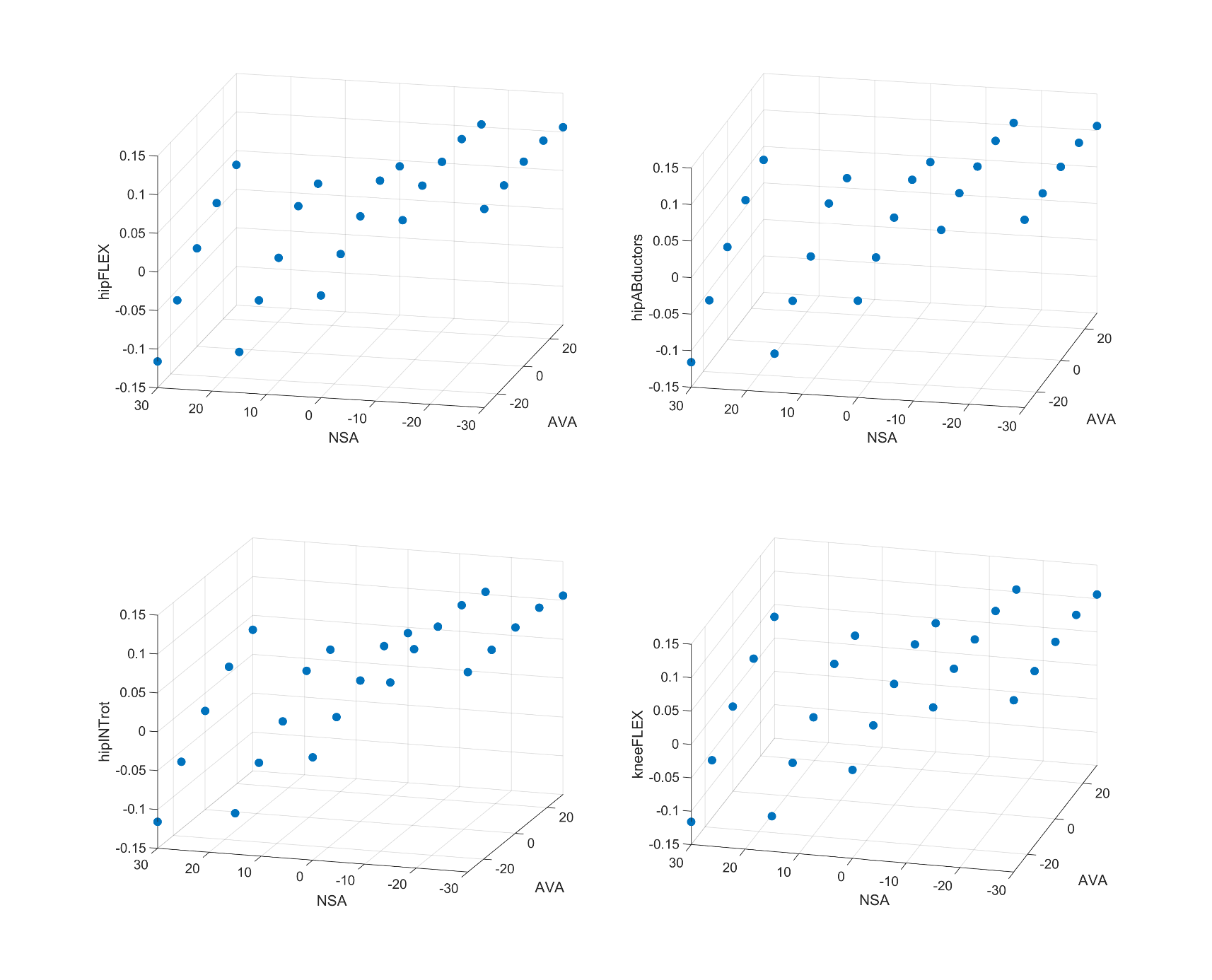


**Figure S20.** Scatterplots showing the relationship between the femoral geometry (AVA and NSA) and CCI.


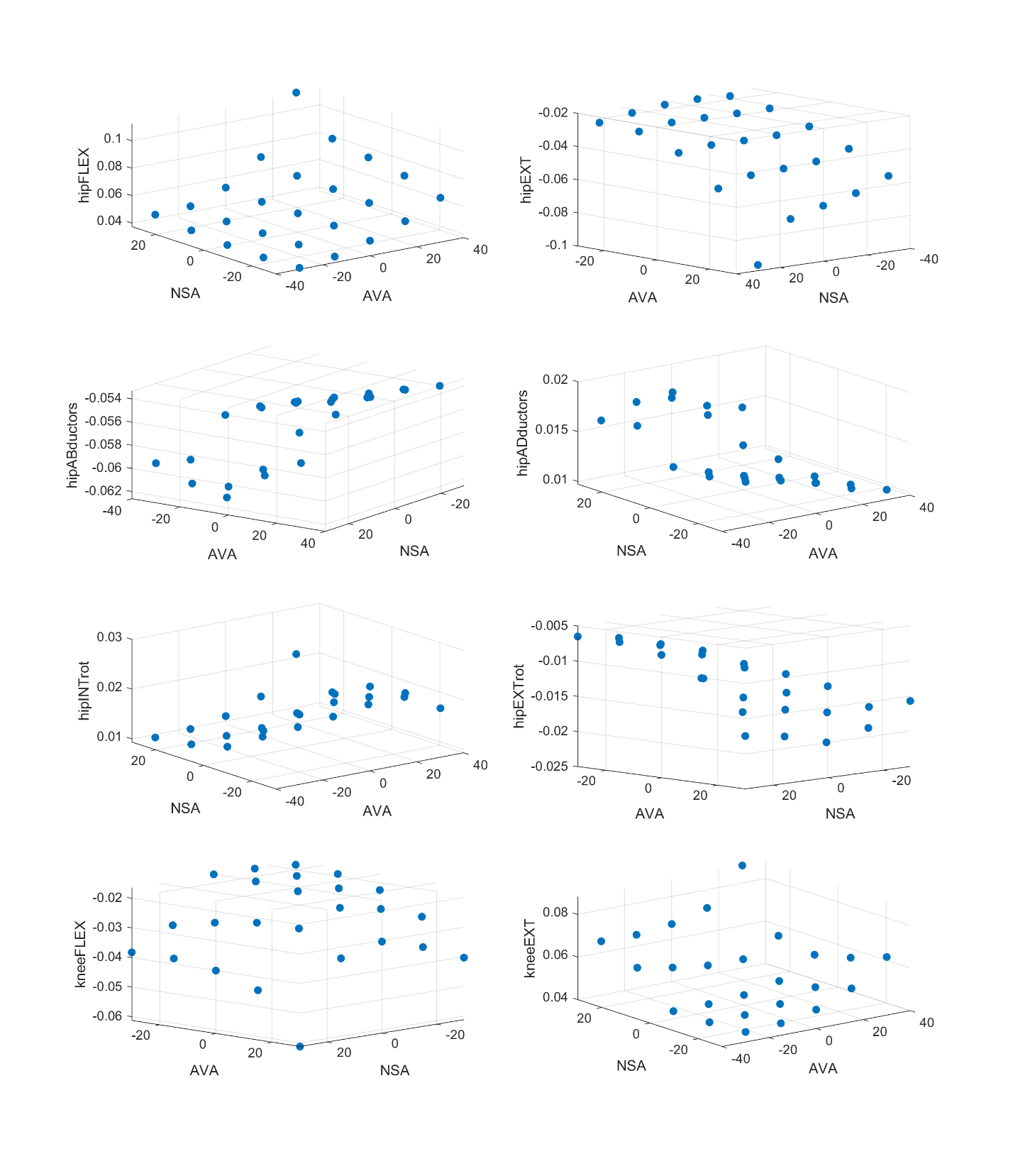


**Figure S21.** Scatterplots showing the relationship between the femoral geometry (AVA and NSA) and muscle moments.
