# Supplementary material for: Influence of femoral anteversion angle and neck-shaft angle on muscle forces and joint loading during walking
Source: PLoS One. 2023 Oct 12;18(10):e0291458. doi: 10.1371/journal.pone.0291458 (PMC10569567; doi:10.1371/journal.pone.0291458)
Supplement: S2 File — (DOCX) [file pone.0291458.s002.docx]

Supplementary material to accompany: Influence of femoral anteversion angle and neck-shaft angle on muscle forces and joint loading during walking

Hans Kainz^1^*, Gabriel T. Mindler^2,3^, Andreas Kranzl^3,4^

^1^ Centre for Sport Science and University Sports, Department of Biomechanics, Kinesiology and Computer Science in Sport, Neuromechanics Research Group, University of Vienna, Vienna, Austria

^2^ Department of Pediatric Orthopaedics, Orthopaedic Hospital Speising, Vienna, Austria

^3^Vienna Bone and Growth Center, Vienna, Austria

^4^Laboratory for Gait and Movement Analysis, Orthopaedic Hospital Speising, Vienna, Austria

# * corresponding author: Ass-Prof. Dr. Hans Kainz, hans.kainz@univie.ac.at

Content of this document: Verification of model modifications and simulation results

VERIFICATION OF SMOOTH MUSCLE MOMENT AND MUSCLE LENGTH WAVEFORMS

Below are examples of the quality control plots which we used to check if muscle moment arm and muscle length waveforms were smooth. A Matlab script called checkMuscleMomentArms.m is freely available on <https://simtk.org/projects/torsiontool>, which can be used to perform the quality check in a fast and easy way. The used models and obtained kinematic results necessary for the quality check are freely available on <https://simtk.org/projects/bone_gait_load>.


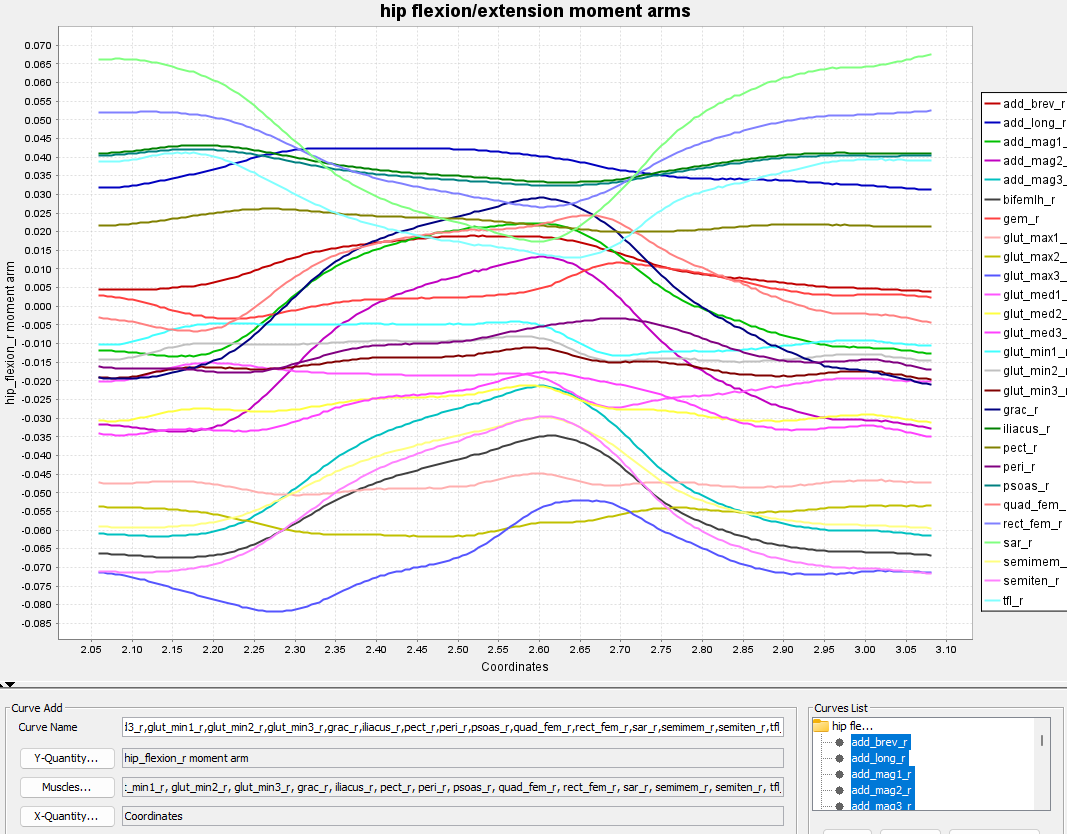


**Figure S22.** Hip flexion/extension moment arms during the evaluated gait cycle.


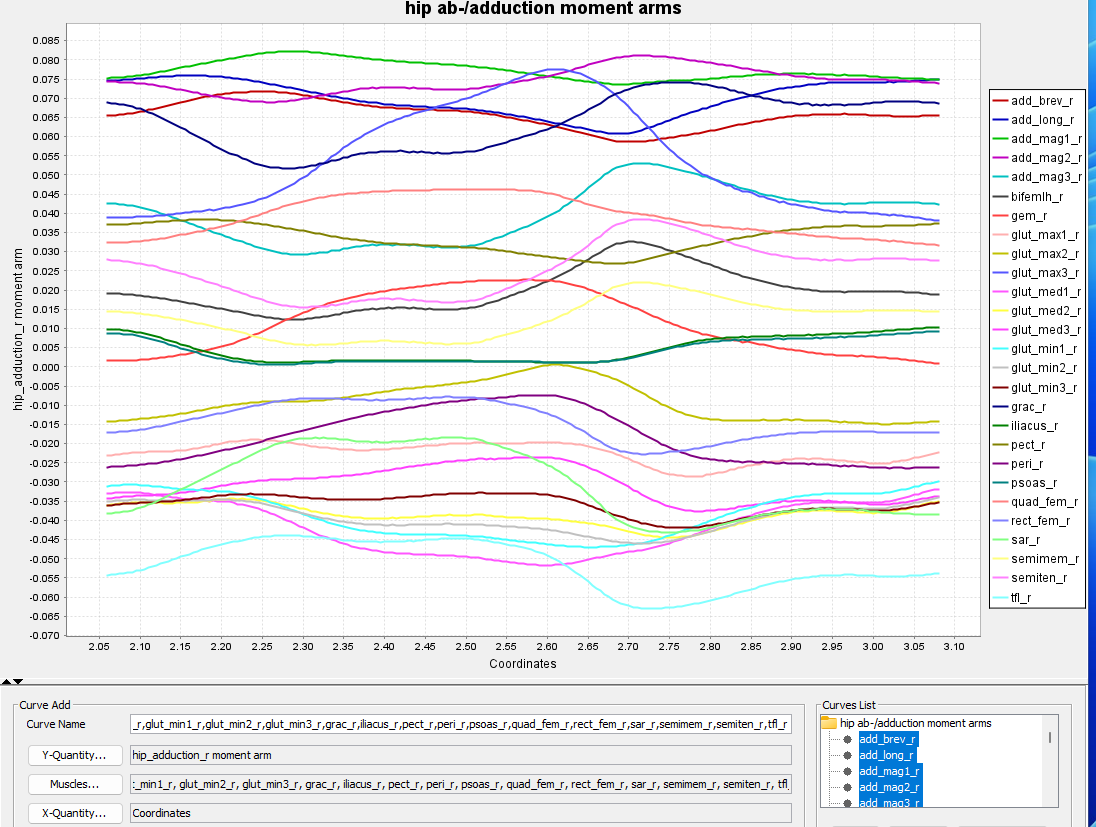


**Figure S23.** Hip ab-/adduction moment arms during the evaluated gait cycle.


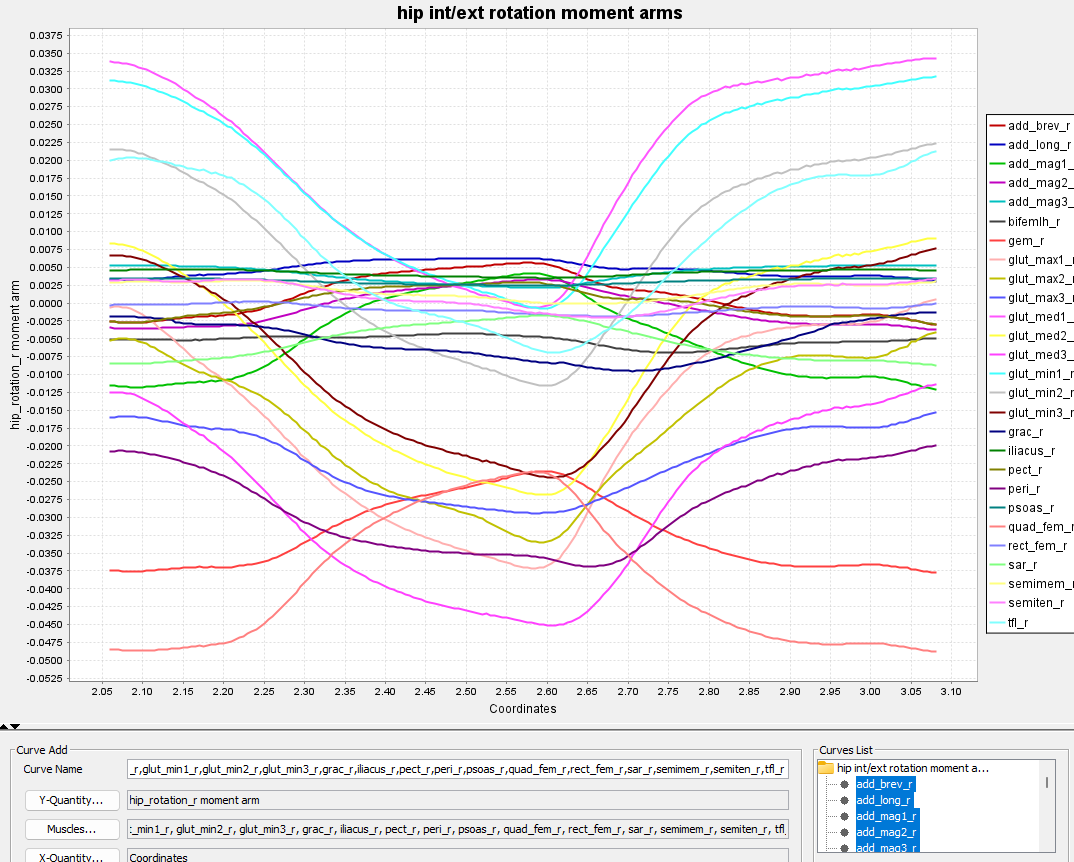


**Figure S24.** Hip internal/external rotation moment arms during the evaluated gait cycle.


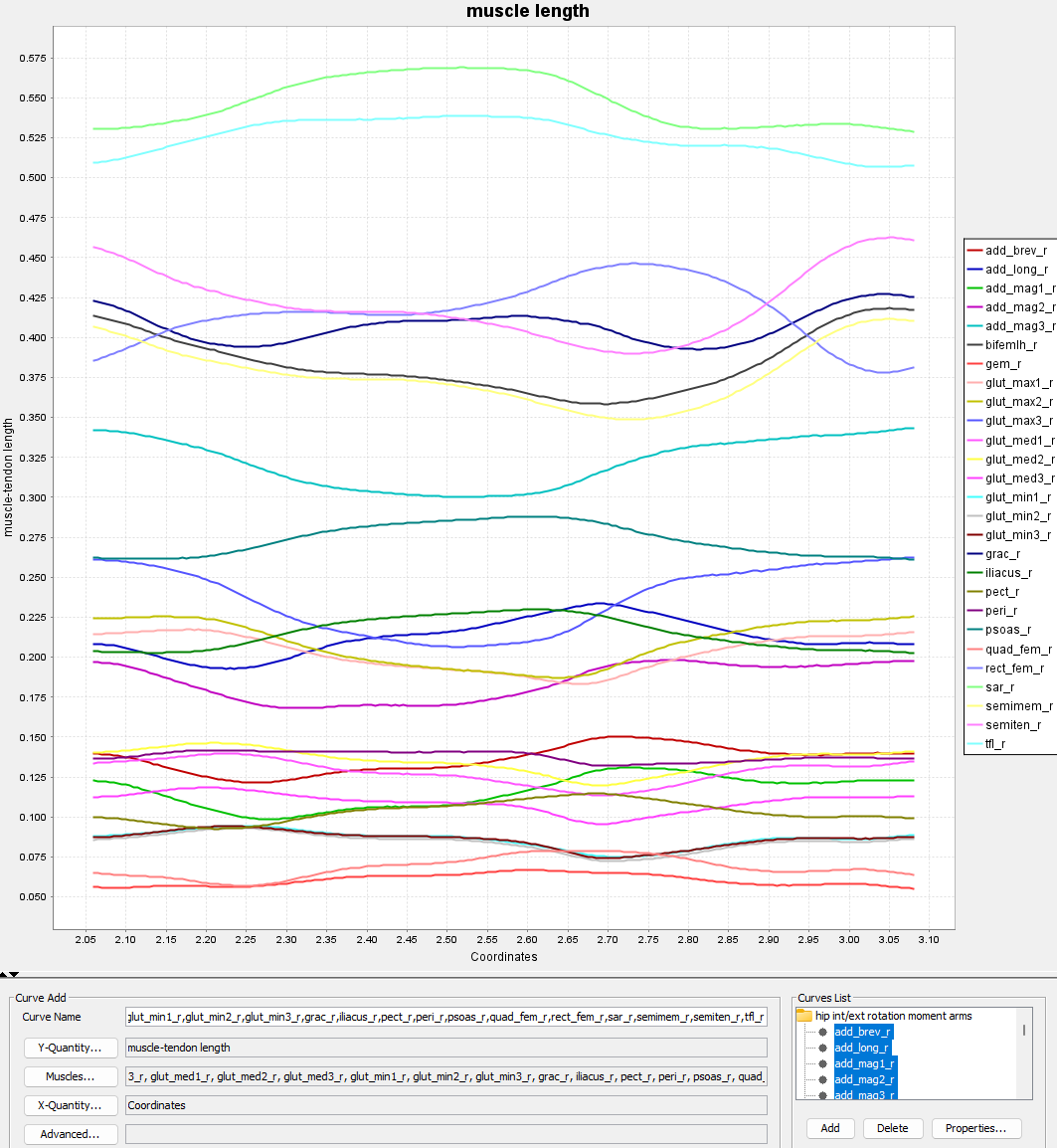


**Figure S25.** Muscle length during the evaluated gait cycle.

MUSCLE ACTIVATION AND FORCES WAVEFORMS


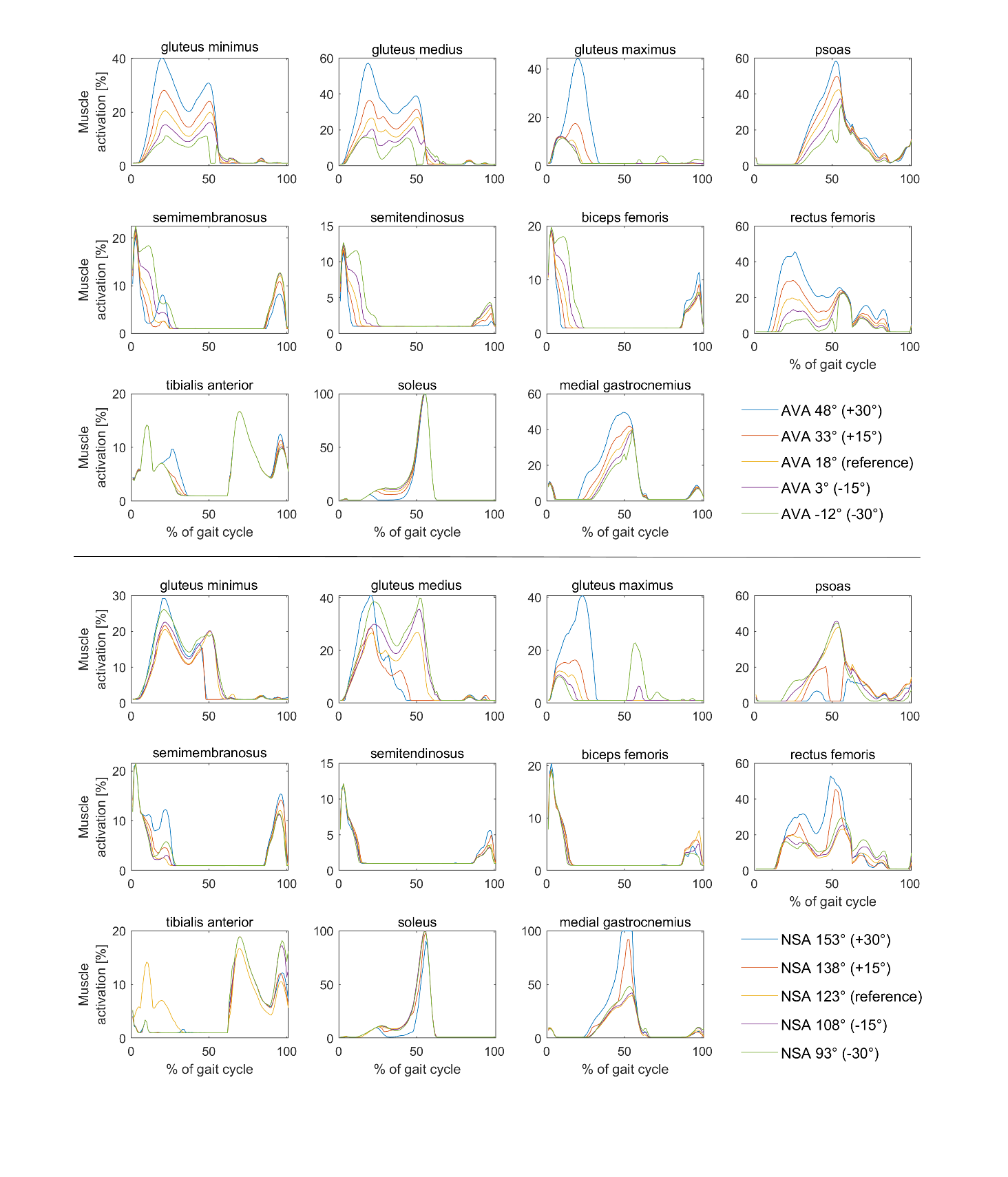


**Figure S26.** Muscle activation waveform for a selection of muscles. Muscle activation waveform showed a reasonable agreement with experimentally measured electromyography signals ^1,2^.


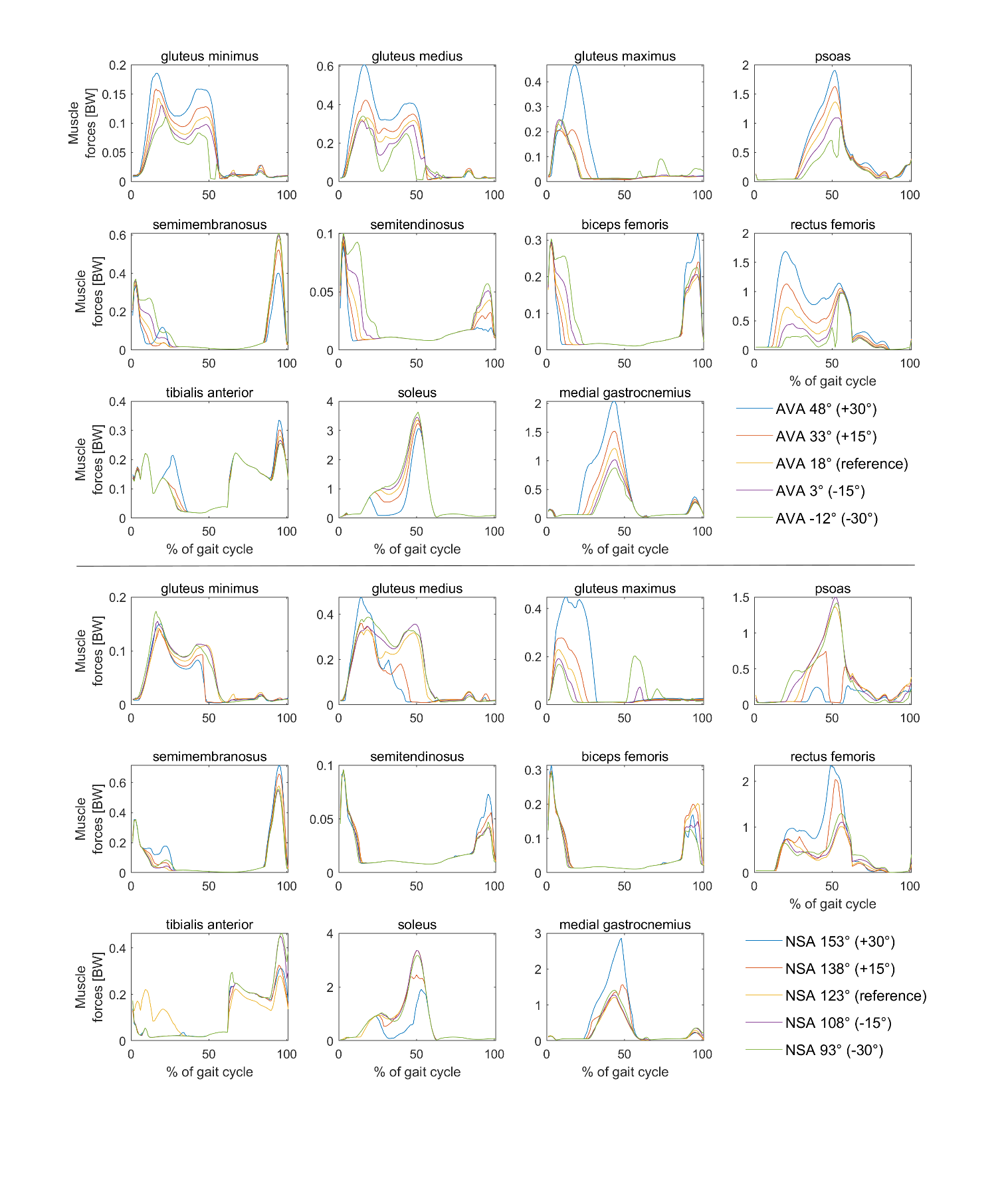


**Figure S27.** Muscle force waveform for a selection of muscles. Muscle force waveform showed a reasonable agreement with previously estimated muscle forces ^3,4^.

**References**

1. Sutherland, D. H. The evolution of clinical gait analysis part l: kinesiological EMG. *Gait Posture* **14**, 61–70 (2001).

2. Agostini, V. *et al.* Normative EMG activation patterns of school-age children during gait. *Gait Posture* **32**, 285–289 (2010).

3. De Pieri, E., Cip, J., Brunner, R., Weidensteiner, C. & Alexander, N. The functional role of hip muscles during gait in patients with increased femoral anteversion. *Gait Posture* **100**, 179–187 (2023).

4. Kainz, H. *et al.* Selective dorsal rhizotomy improves muscle forces during walking in children with spastic cerebral palsy. *Clin. Biomech. (Bristol, Avon)* **65**, 26–33 (2019).
