# Supplementary material for: Influence of femoral anteversion angle and neck-shaft angle on muscle forces and joint loading during walking
Source: PLoS One. 2023 Oct 12;18(10):e0291458. doi: 10.1371/journal.pone.0291458 (PMC10569567; doi:10.1371/journal.pone.0291458)
Supplement: Attachment — Submitted filename: PONE-D-23-11896_reviewer_comments.pdf [file pone.0291458.s003.pdf]

PLOS ONE

Influence of femoral anteversion angle and neck-shaft angle on muscle forces and joint loading during walking

--Manuscript Draft--

**Manuscript Number:**	PONE-D-23-11896
**Article Type:**	Research Article
**Full Title:**	Influence of femoral anteversion angle and neck-shaft angle on muscle forces and joint loading during walking
**Short Title:**	Influence of femoral geometry on muscle forces and joint loading during walking
**Corresponding Author:**	Hans Kainz<br>Universitat Wien<br>Vienna, AUSTRIA
**Keywords:**	musculoskeletal model; femoral geometry; muscle forces; anteversion angle; gait analysis; joint contact forces
**Abstract:**	Femoral deformities, e.g. increased or decreased femoral anteversion (AVA) and neck-shaft angle (NSA), can lead to pathological gait patterns, altered joint loads, and degenerative joint diseases. The mechanism how femoral geometry influences muscle forces and joint load during walking is still not fully understood. The objective of our study was to investigate the influence of femoral AVA and NSA on muscle forces and joint loads during walking.<br>We conducted a comprehensive musculoskeletal modelling study based on three-dimensional motion capture data of a healthy person with a typical gait pattern. We created 25 musculoskeletal models with a variety of NSA (93°-153°) and AVA (-12°-48°). For each model we calculated moment arms, muscle forces, muscle moments, co-contraction indices and joint loads using OpenSim. Multiple regression analyses were used to predict muscle activations, muscle moments, co-contraction indices, and joint contact forces based on the femoral geometry.<br>We found a significant increase in co-contraction of hip and knee joint spanning muscles in models with increasing AVA and NSA, which led to a substantial increase in hip and knee joint contact forces. Decreased AVA and NSA had a minor impact on muscle and joint contact forces. Large AVA lead to increases in both knee and hip contact forces. Large NSA (153°) combined with large AVA (48°) led to increases in hip joint contact forces by five times body weight. Low NSA (108° and 93°) combined with large AVA (48°) led to two-fold increases in the second peak of the knee contact forces. Increased joint contact forces in models with increased AVA and NSA were linked to changes in hip muscle moment arms and compensatory increases in hip and knee muscle forces. Knowing the influence of femoral geometry on muscle forces and joint loads can help clinicians to improve treatment strategies in patients with femoral deformities.
**Order of Authors:**	Hans Kainz
	Gabriel T. Mindler
	Andreas Kranzl
**Additional Information:**	
**Question**	**Response**
**Financial Disclosure**<br><br>Enter a financial disclosure statement that describes the sources of funding for the work included in this submission. Review the submission guidelines for detailed requirements. View published research articles from *PLOS ONE* for specific	The authors received no specific funding for this work.

Powered by Editorial Manager® and ProduXion Manager® from Aries Systems Corporation

examples.<br><br>This statement is required for submission and **will appear in the published article** if the submission is accepted. Please make sure it is accurate.<br><br>**Unfunded studies**<br>Enter: *The author(s) received no specific funding for this work.*<br><br>**Funded studies**<br>Enter a statement with the following details:<br>• Initials of the authors who received each award<br>• Grant numbers awarded to each author<br>• The full name of each funder<br>• URL of each funder website<br>• Did the sponsors or funders play any role in the study design, data collection and analysis, decision to publish, or preparation of the manuscript?<br>• **NO** - Include this sentence at the end of your statement: *The funders had no role in study design, data collection and analysis, decision to publish, or preparation of the manuscript.*<br>• **YES** - Specify the role(s) played.<br><br>* typeset	
**Competing Interests**<br><br>Use the instructions below to enter a competing interest statement for this submission. On behalf of all authors, disclose any competing interests that could be perceived to bias this work—acknowledging all financial support and any other relevant financial or non-financial competing interests.<br><br>This statement is required for submission and **will appear in the published article** if the submission is accepted. Please make sure it is accurate and that any funding sources listed in your Funding Information later in the submission form are also declared in your Financial Disclosure statement.	The authors have declared that no competing interests exist.

Powered by Editorial Manager® and ProduXion Manager® from Aries Systems Corporation

View published research articles from *PLOS ONE* for specific examples.<br><br>**NO authors have competing interests**<br><br>Enter: *The authors have declared that no competing interests exist.*<br><br>**Authors with competing interests**<br><br>Enter competing interest details beginning with this statement:<br><br>*I have read the journal's policy and the authors of this manuscript have the following competing interests: [insert competing interests here]*<br><br>* typeset	
**Ethics Statement**<br><br>Enter an ethics statement for this submission. This statement is required if the study involved:<br><br>• Human participants<br>• Human specimens or tissue<br>• Vertebrate animals or cephalopods<br>• Vertebrate embryos or tissues<br>• Field research<br><br>Write "N/A" if the submission does not require an ethics statement.<br><br>General guidance is provided below. Consult the submission guidelines for detailed instructions. **Make sure that all information entered here is included in the Methods section of the manuscript.**	All methods were carried out in accordance with the relevant guidelines and regulations. The used dataset for our simulations was part of a bigger project approved by our local ethics committee (Number: EK20/2022, Ethikkommission der Wiener Häuser der Vinzenz Gruppe, Vienna, Austria) for capturing reference data for the gait laboratory database of the Orthopedic Hospital Speising (Vienna, Austria). Signed written informed consent to use the collected data for the laboratory reference database and scientific studies was obtained from the participant prior to the measurements. Participants were recruited and data was collected between August 2022 and October 2022. Data processing and simulation for the current study were performed between November 2022 and February 2023. AK selected the data set for this study. He was the only person who had access to information that could identify the participant. All further processing and musculoskeletal simulations were performed based on the anonymized data set.

Powered by Editorial Manager® and ProduXion Manager® from Aries Systems Corporation

**Format for specific study types**<br><br>**Human Subject Research (involving human participants and/or tissue)**<br>• Give the name of the institutional review board or ethics committee that approved the study<br>• Include the approval number and/or a statement indicating approval of this research<br>• Indicate the form of consent obtained (written/oral) or the reason that consent was not obtained (e.g. the data were analyzed anonymously)<br><br>**Animal Research (involving vertebrate animals, embryos or tissues)**<br>• Provide the name of the Institutional Animal Care and Use Committee (IACUC) or other relevant ethics board that reviewed the study protocol, and indicate whether they approved this research or granted a formal waiver of ethical approval<br>• Include an approval number if one was obtained<br>• If the study involved *non-human primates*, add *additional details* about animal welfare and steps taken to ameliorate suffering<br>• If anesthesia, euthanasia, or any kind of animal sacrifice is part of the study, include briefly which substances and/or methods were applied<br><br>**Field Research**<br><br>Include the following details if this study involves the collection of plant, animal, or other materials from a natural setting:<br>• Field permit number<br>• Name of the institution or relevant body that granted permission	
**Data Availability**<br><br>Authors are required to make all data underlying the findings described fully available, without restriction, and from the time of publication. PLOS allows rare exceptions to address legal and ethical concerns. See the PLOS Data Policy and FAQ for detailed information.	Yes - all data are fully available without restriction

A Data Availability Statement describing where the data can be found is required at submission. Your answers to this question constitute the Data Availability Statement and **will be published in the article**, if accepted.<br><br>**Important:** Stating 'data available on request from the author' is not sufficient. If your data are only available upon request, select 'No' for the first question and explain your exceptional situation in the text box.<br><br>Do the authors confirm that all data underlying the findings described in their manuscript are fully available without restriction?	
**Describe where the data may be found in full sentences. If you are copying our sample text, replace any instances of XXX with the appropriate details.**<br><br>• If the data are **held or will be held in a public repository**, include URLs, accession numbers or DOIs. If this information will only be available after acceptance, indicate this by ticking the box below. For example: *All XXX files are available from the XXX database (accession number(s) XXX, XXX.)*.<br>• If the data are all contained **within the manuscript and/or Supporting Information files**, enter the following: *All relevant data are within the manuscript and its Supporting Information files.*<br>• If neither of these applies but you are able to provide **details of access elsewhere**, with or without limitations, please do so. For example:<br><br>*Data cannot be shared publicly because of [XXX]. Data are available from the XXX Institutional Data Access / Ethics Committee (contact via XXX) for researchers who meet the criteria for access to confidential data.*<br><br>*The data underlying the results presented in the study are available from (include the name of the third party*	Data related to this study is freely available on https://simtk.org/projects/bone_gait_load

and contact information or URL).<br>• This text is appropriate if the data are owned by a third party and authors do not have permission to share the data.<br><br>* typeset	
Additional data availability information:	

Powered by Editorial Manager® and ProduXion Manager® from Aries Systems Corporation

Manuscript

Click here to access/download;Manuscript;Manuscript_updated.docx

Influence of femoral anteversion angle and neck-shaft angle on muscle forces and joint loading during walking

Hans Kainz[1]*, Gabriel T. Mindler[2,3], Andreas Kranzl[3,4]

[1] Centre for Sport Science and University Sports, Department of Biomechanics, Kinesiology and Computer Science in Sport, Neuromechanics Research Group, University of Vienna, Vienna, Austria

[2] Department of Pediatric Orthopaedics, Orthopaedic Hospital Speising, Vienna, Austria

[3]Vienna Bone and Growth Center, Vienna, Austria

[4]Laboratory for Gait and Movement Analysis, Orthopaedic Hospital Speising, Vienna, Austria

* corresponding author: Ass-Prof. Dr. Hans Kainz, hans.kainz@univie.ac.at

Abstract

Femoral deformities, e.g. increased or decreased femoral anteversion (AVA) and neck-shaft angle (NSA), can lead to pathological gait patterns, altered joint loads, and degenerative joint diseases. The mechanism how femoral geometry influences muscle forces and joint load during walking is still not fully understood. The objective of our study was to investigate the influence of femoral AVA and NSA on muscle forces and joint loads during walking.

We conducted a comprehensive musculoskeletal modelling study based on three-dimensional motion capture data of a healthy person with a typical gait pattern. We created 25 musculoskeletal models with a variety of NSA (93°-153°) and AVA (-12°-48°). For each model we calculated moment arms, muscle forces, muscle moments, co-contraction indices and joint loads using OpenSim. Multiple regression analyses were used to predict muscle activations, muscle moments, co-contraction indices, and joint contact forces based on the femoral geometry.

We found a significant increase in co-contraction of hip and knee joint spanning muscles in models with increasing AVA and NSA, which led to a substantial increase in hip and knee joint contact forces. Decreased AVA and NSA had a minor impact on muscle and joint contact forces. Large AVA lead to increases in both knee and hip contact forces. Large NSA (153°) combined with large AVA (48°) led to increases in hip joint contact forces by five times body weight. Low NSA (108° and 93°) combined with large AVA (48°) led to two-fold increases in the second peak of the knee contact forces. Increased joint contact forces in models with increased AVA and NSA were linked to changes in hip muscle moment arms and compensatory increases in hip and knee muscle forces. Knowing the influence of femoral geometry on muscle forces and joint loads can help clinicians to improve treatment strategies in patients with femoral deformities.

Introduction

Torsional deformities of lower limbs are common in patients with and without neurological disorders [1,2], and are a frequent reason for consultation in pediatric orthopedics [3]. Many idiopathic torsional deformities seen in daily practice are minor and have little clinical significance. However, excessive malalignment can lead to an altered gait pattern, increased risk of falls, functional limitations, overuse injuries, joint pain and increased risk for clinical problems such as osteoarthritis [4–8].

The femoral anteversion and neck-shaft angle are two important anatomical features of the femur [9]. Anteversion angle (AVA) is the angle in the transverse plane by which the neck of the femur deviates forwards from the axis of the femoral condyles. The neck-shaft angle (NSA) is the angle between the neck and the shaft of the femur. In typically developing children, femoral AVA decreases from approximately 40° at birth to 15° at skeletal maturity, whereas the NSA decreases from 140° to 125°. Torsional femoral deformities are defined as an increased or decreased AVA of a patient compared to age-matched typically developing children and occur in children with and without neurological disorders. In some individuals the AVA and NSA barely change during childhood and therefore with time develop large differences to the values of typically developing children [1,10,11]. In healthy adults, typical AVA and NSA are between 10° and 20° and between 124° and 136°, respectively [12,13].

Femoral deformities can lead to a pathological gait pattern. In patients with idiopathic internal torsional deformities Mackay et al. [4] observed an increased internal hip rotation and decreased foot progression angle. Furthermore, they reported a high prevalence of pain in children with torsional deformities, especially at the hip (63%) and knee (58%) joints [4]. Uemura et al. [14] found a strong positive correlation (r=0.8) between the femoral AVA and hip rotation during gait, which was partly contrary to the findings from Radler et al. [3], who only found a weak correlation (r=0.2) between AVA and hip rotation in children with idiopathic torsional deformities. Passive hip rotation has been shown to be a predictor for femoral torsion [15] and in-toing gait [16]. Bruderer-Hofstetter et al. [17] found a greater knee adduction, greater anterior pelvic tilt and more flexed hips in children with idiopathic torsional

deformities. Alexander et al. [2] showed that children with increased AVA walk with increased knee flexion and increased frontal plane hip and knee moments. In children with cerebral palsy, Braatz et al. [18] showed that a de-rotation osteotomy to normalize the AVA also alters the foot progression angle and hip rotation during walking. In summary, it has been shown that femoral deformities lead to pathological gait pattern in many but not all individuals. The observed pathological gait pattern in patients with torsional deformities are very heterogeneous, potentially due to different compensation strategies. So far, it is not clear why some individuals with femoral deformities walk with a typical gait pattern whereas others use a pathological gait pattern. Furthermore, it is unknown how the NSA influences the ability to walk normal.

Musculoskeletal modelling has been used to increase our insights in torsional deformities for more than two decades. The early work from Scott Delp's research group (Stanford University) showed that increasing the AVA decreased the abduction moment arm of the gluteus medius muscles but internal rotation of the hip restores the moment arm [19]. Furthermore, they showed that de-rotation osteotomies barely change the lengths of the hamstrings, gracilis and adductor muscles [20]. In children with cerebral palsy and internally-rotated gait the medial hamstrings, adductor brevis and gracilis muscles have a negligible internal rotation moment arm and therefore are unlikely to contribute to the internal rotated gait [21]. Increasing AVA shifted the moment arms from the hamstrings and adductors towards external rotation and therefore these muscles are unlikely to cause the internal rotated gait in children with cerebral palsy [22]. Additional early work conducted by Ilse Jonker's research group (KU Leuven) showed that a patient-specific gait pattern in children with cerebral palsy and increased AVA beneficially increased the ability of the gluteus medius and maximus to extend the hip and knee, whereas the potential of the hamstrings to extend the hip was decreased [23]. In another paper they showed that the patient-specific gait pattern reduces hip loading in children with increased AVA [24]. Heller et al. [25] showed that increasing the AVA increases hip joint contact forces when tracking the same, subject-specific motion capture data of four patients with a total hip arthroplasty, which was confirmed by recent simulation studies [26–28]. Increased hip and patellofemoral loading was found in

children with increased AVA and normal foot progression angle [29], whereas decreased hip and knee loads were observed in children with increased AVA and internal foot progression angle compared to healthy control participants [30]. In summary, previous studies showed that increased AVA alters the moment arms of certain muscles and leads to increased hip and knee joint loads, which can be compensated with an altered gait pattern. However, it remains unclear how the subject-specific gait pattern combined with altered moment arms influence muscle forces and the generated moments by each muscle and therefore alter joint loads. Furthermore, it is unknown how the NSA influences and contributes to the altered muscle forces and joint loading.

The aim of our study was to comprehensively investigate the influence of femoral AVA and NSA on muscle forces and joint loads during walking. Investigating the influence of femoral geometry on joint loads is challenging with conventional approaches because each person has a subject-specific femoral AVA and NSA, and walks with an individual walking pattern, i.e. joint angles and walking velocity. Hence, numerous variables would affect the estimated joint loads. To overcome this limitation, we decided to conduct a series of 'what-if' simulations, which enabled us to keep all variables constant and isolate the impact of altered femoral geometry on muscle forces and joint loads. We created 25 musculoskeletal models with a variety of NSA and AVA. For each model we calculated moment arms, muscle forces and joint loads based on the motion capture data of a person with a typical gait pattern. We hypothesized that the increased joint loads in models with increasing AVA are caused by increased co-contraction of hip and knee spanning muscles. Based on previous research [26], we furthermore hypothesized that models with increasing NSA will increase co-contraction and joint loads. Knowing the influence of femoral geometry on muscle forces and joint loads during walking could help clinicians to improve treatment strategies in patients with femoral deformities.

Methods

Musculoskeletal models

The gait2392 musculoskeletal OpenSim model [31,32] was used as the reference model for our simulations. The model included three degrees of freedom at each hip joint, one degree of freedom at each knee and ankle joint and three degrees of freedom between the pelvis and torso segment. Furthermore, the model included 92 muscle-tendon units, representing the muscles in the lower extremities and torso. The femur in the reference model had an NSA of 123° and an AVA of 18°. To investigate the impact of proximal femoral geometry on muscle forces and joint loads in a systematic way we created 24 additional models with varying NSA (±30° in 15° steps, absolute NSA between 93° and 153°) and AVA (±30° in 15° steps, absolute AVA between -12° and 48°). The recently developed and published Torsion Tool [33] to personalize bony geometries in OpenSim models was used to generate the additional models. Briefly described, the tool changes the vertices of the femur based on pre-defined boxes to match the chosen NSA and AVA. This procedure alters all the muscle origin and insertion points within the boxes. The tool altered the proximal femoral geometry and therefore did not influence the anatomical coordinate system of the femur.

Motion capture data

All simulations were based on three-dimensional gait analysis data, i.e. marker trajectories and ground reaction forces, obtained during barefoot walking on an instrumented walkway from a healthy person (weight: 73.1 kg, height: 1.71 m, walking velocity: 1.41 m/s) without any known abnormalities that could have altered the participant's gait pattern. Data was collected with a modified Cleveland marker set for the lower extremities and a Plug in Gait marker set for the upper extremities [34]. All methods were carried out in accordance with the relevant guidelines and regulations. The used dataset for our simulations was part of a bigger project approved by our local ethics committee (Number: EK20/2022, Ethikkommission der Wiener Häuser der Vinzenz Gruppe, Vienna, Austria) for capturing reference data for the gait laboratory database of the Orthopedic Hospital Speising (Vienna, Austria). Signed written informed consent to use the collected data for the laboratory reference database and scientific studies was obtained from the participant prior to the measurements. Participants were recruited and data was collected between August 2022 and October 2022. Data processing and simulation for the

current study were performed between November 2022 and February 2023. AK selected the data set for this study. He was the only person who had access to information that could identify the participant. All further processing and musculoskeletal simulations were performed based on the anonymized data set.

Musculoskeletal simulations

Each musculoskeletal model was scaled to the anthropometry of our participant based on the location of surface markers and estimated joint centres [35]. Optimal fiber lengths and tendon slack lengths of each muscle were optimized to fit to the each scaled model using the Matlab tool developed by Modenese et al. [36]. Muscle activations of the reference model (NSA of 123° and AVA of 18°) led to unrealistic high values, i.e. 100% activation for several muscles. Hence, the maximum isometric force of each muscle was multiplied by two to allow the generation of realistic muscle activation waveforms with the reference model, i.e. avoid plateaus of 100% muscle activation. Increasing maximum isometric muscle forces of the gait2392 model is a common practice when analyzing movements of healthy, young adults [37,38] because the original force values are based on data from elderly specimens and therefore are not representative for our participant. Inverse kinematics and inverse dynamics were used to calculate joint angles and moments, respectively. Muscle forces were estimated using static optimization while minimizing the sum of squared muscle activations and accounting for the muscle force-length-velocity relationship [39]. Afterwards, joint reaction load analysis [40] was performed to calculate hip, knee, and ankle joint contact forces. OpenSim's analyze tool was used to obtain the muscles' moment arms and muscle-tendon lengths for each model. All simulations were performed in OpenSim 4.2.

Data analyses

All simulation results were normalized to 100% of the gait cycle. Additionally, joint moments were normalized to body mass, and muscle forces and joint contact forces were normalized to body weight. The moment each muscle generates was calculated by multiplying the muscle forces with the moment

arm in each anatomical plane for each time frame during the gait cycle. To quantify agonist ($M_{agonist}$) and antagonist muscle moments ($M_{antagonist}$) the sum of muscle moments for each anatomical plane and direction was calculated. These muscle moments are referred to hip flexors and extensors, hip abductors and adductors, hip internal and external rotators, knee flexors and extensors muscle moments in our manuscript. Similar to previous studies [41], we calculated the co-contraction index (CCI) based on the muscle moments to quantify the amount of co-contraction (Equation 1). CCI values of 0, 1 and -1 indicate full co-contraction, only antagonist activation, and only agonist activation, respectively. Considering that the CCI does not give us a value for the magnitude of co-contraction, we additionally compared the muscle moments between the corresponding agonist and antagonist muscle groups. To address our hypotheses, i.e. increased NSA and AVA increase co-contraction and joint contact forces, we used a multiple regression analysis to predict the mean muscle activations, muscle moments, CCI, and joint contact forces during the stance phase of gait based on the femoral geometry, i.e. NSA and AVA. Statistical significance was set at alpha = 0.05. Additionally, we used descriptive statistics to compare joint angles, joint moments, muscles' moment arms and muscles' lengths between the different models.

$$CCI = \begin{cases} 1 - \frac{M_{agonist}}{M_{antagonist}}, if\ M_{antagonist} > M_{agonist} \\ \frac{M_{antagonist}}{M_{agonist}} - 1, otherwise \end{cases} \qquad \text{Equation 1}$$

Results

Joint angles and moments

Joint angles and moments obtained from our simulations were identical between the different models and comparable to previous studies based on healthy individuals [42] (Fig. 1).

Joint contact forces

As expected, hip and knee joint contact forces increased with increasing NSA and AVA (Fig. 2 and 3). The femoral geometry significantly predicted hip (R^2=0.86, p<0.001) and knee (R^2=0.60, p<0.001) JCF. Both variables, i.e. AVA and NSA, added statistically significantly (p<0.001) to the prediction.

Co-contraction and muscle moments

Increased AVA increased hip flexor/extensor co-contraction and increased hip flexor, hip extensor, knee flexor and knee extensor muscle moments (Fig. 4 and 5). Increased NSA increased knee flexor and knee extensor muscle moments and decreased hip internal and external muscle moments.

The femoral geometry significantly predicted hip flexor/extensor CCI (R^2=0.92, p<0.001), hip ab-/adductor CCI (R^2=0.47, p<0.001), hip internal/external rotator CCI (R^2=0.84, p<0.001) and knee flexor/extensor CCI (R^2=0.27, p=0.033). Both variables, i.e. AVA and NSA, added statistically significantly (p<0.05) to the prediction of hip ab-/adductor CCI and hip internal/external rotator CCI, whereas only AVA was a significant predictor (p<0.001) for hip flexor/extensor CCI and only NSA was a significant predictor (p<0.01) for knee flexor/extensor CCI.

The femoral geometry significantly predicted all analyzed muscle moments (R^2 between 0.57 and 0.84, p<0.001 for all moments). Both variables, i.e. AVA and NSA, added statistically significantly (p<0.05) to the prediction of muscle moments except for hip internal and external muscle moments, where only the NSA was a significant predictor.

Muscle activations

Increasing AVA increased muscle activation of all hip and knee spanning muscle groups (Fig 6). Increasing NSA altered muscle activations of all hip and knee spanning muscle groups except for hip flexor, hip external rotators and knee extensor muscles.

The femoral geometry significantly predicted muscle activations of all analyzed muscle groups (R^2 between 0.54 and 0.82, p<0.001 for all muscle groups). Both variables, i.e. AVA and NSA, added

statistically significantly ($p<0.05$) to the prediction of muscle activations except for hip flexor, hip external rotators and knee extensor muscles, where only the AVA was a significant predictor.

Moment arms and muscle forces

Average moment arms of hip extensor muscles increased with increasing AVA, whereas moment arms of hip flexor muscles barely changed (Fig. 7). Both, hip flexor and extensor muscle forces increased with increasing AVA but the increase in muscle forces was higher for hip flexor compared to hip extensor muscles (Fig. 8).

Increased AVA decreased the hip abduction moment arms of the majority of hip abductor muscles (Fig. 7), i.e. gluteus minimus, medius, and maximus, tensor fasciae latae, and piriformis. None of the hip abductor moment arms increased with increasing AVA. Increasing NSA led to an additional decrease in hip abduction moment arms. Both, hip abductor and adductor muscle forces increased with increasing AVA but the increase in muscle forces was higher for hip abductor compared to hip adductor muscles (Fig. 8).

Average moment arms of hip internal and external rotator muscles slightly decreased with increasing AVA, whereas muscle forces increased with increasing AVA. Increasing NSA decreased hip internal rotator moment arms (Fig. 7 and 8). No change in knee flexion/extension moment arms were observed between models but increasing the AVA led to increased knee flexor and extensor muscle forces.

Muscle-tendon length

For the majority of muscles, the mean muscle-tendon lengths during the stance phase of the gait cycle barely changed with the altered femoral geometry (supplementary figure S1). A small decrease in muscle-tendon length with increasing AVA was found for the gluteus maximus, quadratus femoris, gemellus and piriformis muscle.

Verification of simulation results

The simulation from our reference model, i.e. AVA of 18° and NSA of 123°, led to maximum hip, knee and ankle joint contact forces of 5.1 times body weight (BW), 3.6 BW, and 5.7 BW, respectively. The magnitude and shape of the contact force waveforms were in agreement with previous simulation studies [29,43–45]. In-vivo measurements of joint loads based on instrumented implants showed lower hip and knee joint contact forces (maximum values of 3.2 BW) [46] compared to our simulations, potentially due to the different walking velocity [47,48] between the elderly patients with a joint replacement (3-4 km/h) and our young, healthy participant (5.1 km/h). Muscle activations and forces from our simulations showed a reasonable agreement with experimentally measured electromyography signals [49,50] and previously estimated muscle forces [51,52] (supplementary material).

We plotted the moment arms and muscle length from all models, i.e. reference and deformed models, to verify that muscle-tendon kinematics is reasonable, i.e. does not lead to discontinuities during the walking pattern of our participant. For all models, dynamic muscle moment arms and muscle-tendon lengths showed smooth waveform throughout the whole gait cycle of our participant.

Discussion

We comprehensively investigated the influence of femoral AVA and NSA on muscle forces and joint loads during walking. In agreement with our hypotheses, we showed that the AVA and NSA are significant ($p<0.05$) predictors for co-contraction and muscle moments in hip and knee joint spanning muscles, and hip and knee joint contact forces. Increased iliacus, psoas, gluteus, tensor fasciae latae, and rectus femoris forces caused increased hip flexor and extensor muscle moments and explain the increase in hip joint contact forces in models with increasing AVA. In models with increasing NSA, the majority of hip muscles' moment arms decreased and muscle forces increased, which explains the increased hip joint contact forces despite similar or even decreasing agonist and antagonist muscle moments. Increased rectus femoris and gastrocnemius muscle forces were mainly responsible for the

increased knee extensor and flexor muscle moments, which resulted in increased knee joint contact forces in models with increasing AVA and NSA.

Hip and knee joint contact forces significantly increased with increasing AVA and NSA in our study, which confirmed the findings of previous studies [25–27]. Our study, however, was the first study that investigated the combined impact of altered AVA and NSA on muscle forces and joint loads. The highest joint contact forces were observed in the model with 30°increased AVA and NSA, i.e. AVA of 48° and NSA of 153°. Hip and knee joint contact forces in this model increased more than five times body weight compared to the values of the reference model (Fig. 2). Interestingly, only in models with AVA above 18°, increasing and decreasing NSA increased knee joint contact forces. These findings highlight that it is important to account for both, the subject-specific AVA and NSA, when estimating joint contact forces. Neglecting the NSA or AVA, as previously done in some studies [27,30], might lead to errors up to a magnitude of five times body weight and therefore could lead to mis-leading interpretations.

Increasing AVA resulted in a significant increase in co-contraction and muscle moments of hip and knee flexor and extensor muscles, whereas in other hip planes only minor alterations in muscle moments were observed. The increased co-contraction of hip and knee flexor/extensor muscles can be explained by the following cascade:

1. Increased AVA decreased the hip abduction moment arms of the majority of hip abductor muscles (Fig. 7), i.e. gluteus minimus, medius and maximus, tensor fasciae, piriformis. Hence, more muscle forces had to be generated for the majority of hip abductor muscles (Fig. 8) to produce the required hip abduction moment. The increase in hip abductor muscle activations and forces did not increase muscle moments due to the decreased moment arms.
2. The mean hip extension moment arms of the gluteus minimus, medius, and maximus increased with increasing AVA. Considering that the gluteus muscles were increasingly activated to compensate for the reduced hip abduction moment arms, the increased gluteus muscle forces combined with the increased hip extension moment arms produced an increased hip extension moment, which had to be counterbalanced by the antagonist muscles, i.e. hip flexor muscles.

Hip flexor moment arms were barely altered and therefore several hip flexor muscles, i.e. rectus femoris, psoas and iliacus, were increasingly activated and produced higher muscle forces and therefore higher muscle moments and co-contraction with increasing AVA.

3. The rectus femoris, psoas, and iliacus are the strongest hip flexor muscles, i.e. have the largest isometric muscle forces compared to all other hip flexor muscles, and therefore their forces were increased to generate the required hip flexion muscle moment. The rectus femoris is, however, also a knee extensor muscle and therefore produced an additional knee extension moment. This extension moment was counterbalanced by an additional knee flexion moment produced mainly by increased muscle activations and forces of the gastrocnemius (medial and lateral) and short head of the biceps femoris muscles.
4. The increased co-contraction at the hip and knee-spanning muscle highlighted above explains the increased hip and knee joint contact forces in models with increased AVA. Additional figures of moment arms and muscle forces of all individual muscles are provided in the supplementary material and confirm the presented cascade leading to the increased co-contractions.

Large NSA combined with AVA of 48° increased both peaks of the hip joint contact force. Considering that hip flexor/extensor muscle moments did not change, hip ab-/adductor muscle moments only increased during the second half of the stance phase and hip internal/external rotator muscle moments decreased with increasing NSA, muscle moments of hip-spanning muscles cannot fully explain the observed increase in hip joint contact forces. Increasing NSA decreased the moment arm of many agonist and antagonist hip muscles and several muscles (e.g. gluteus medius and maximus) had to produce higher muscle forces to maintain the prescribed joint moments, which likely explains the observed increases in both peaks of hip joint contact forces. In models with large AVA, NSA angles of 153° increased the hip flexor and extensor muscle moments especially during the first half of the stance phase. This additional hip flexor/extensor co-contraction explains the big impact on the first peak of hip joint contact forces in models with increased AVA and NSA (see figures in supplementary material).

Increasing NSA increased knee joint contact forces. Increases in the second peak of knee joint contact forces with increasing NSA were observed in models with low AVA (18° or less) while, in models with large AVA (48°), both peaks of the knee joint contact force increased with increasing NSA from 138° to 153°. The timely concurrent observed increase in knee flexor and extensor muscle moment, i.e. only during the second half of the stance phase in models with low AVA and during the first and second half of the stance phase in models with large AVA, confirm the increased amount of co-contraction responsible for the increased knee joint contact forces (see figures in supplementary material). The rectus femoris and gastrocnemius muscles produced higher forces and were mainly responsible for the increased knee flexor and extensor muscle moments. It seems that the rectus femoris muscle, who's moment arms are not altered with increasing NSA and AVA, has to generate greater forces to compensate for the reduced muscle moments of several hip-spanning muscles due to reduced moment arms.

Joint angles and moment were identical in all our models and comparable to previous studies based on healthy individuals [42,53]. We used the torsion tool [33] to modify the proximal femur in all our models, which did not change the position of the hip joint center, nor did it change the position and orientation of the femoral epicondyles. These anatomical landmarks define the anatomical coordinate system of the femur [54]. Considering that none of these landmarks were altered when modifying the femoral geometries, the anatomical coordinate system of the femur and all other segments remained the same in our models. Furthermore, we used the same linear scaling procedure in all models. Hence, all our models had the same anatomical reference systems and segment lengths, which explains the identical joint angles and moments obtained with our models [55,56].

External joint moments are often used as a surrogate measure for joint contact forces [57,58]. Joint moment estimated via inverse dynamics consider the kinematics of the person and external forces, i.e. ground reaction forces, whereas joint contact forces additionally depend on muscle forces, which are influenced by each muscle's moment arm. Our study highlighted that large differences in joint contact forces, i.e. more than 100% of the reference values or five times body weight, can be caused by different

femoral geometries even if joint kinematics and joint moments are exactly the same. Taking into account the large variability in femoral geometry in children as well as in adults (95% confidence intervals of approximately ±10° and ±20° for NSA and AVA, respectively [59–61]), we suggest not to use joint moments as a surrogate measure for joint contact forces. Furthermore, findings from studies that calculated correlations between joint moments and joint contact forces estimated with musculoskeletal models with generic bones [45,62] should be interpreted with caution.

Several studies [29,63] already suggested that altered joint mechanics were responsible for increased joint loads in people with increased AVA. Our study, however, is the first to comprehensively describe the link between biomechanical parameters and increased joint loads. Femoral geometry and gait pattern can differ between participants, which both influence joint loads [64], and making it difficult to solely quantify the impact of femoral geometry on joint mechanics with traditional experimental studies. Our study based on 'what-if' simulations enabled us to keep gait pattern constant, i.e. joint kinematics and kinetics, and investigate which muscle forces would be required for a typical gait pattern with models with altered femoral geometries. Hence, we could evaluate how the femoral geometry alone influences muscle and joint contact forces due to altered moment arms without any confounding factors.

Many people with altered femoral geometry walk with a pathological gait pattern [2–4,14,17], which was neglected in our study. Nevertheless, our simulation results might help to explain why some people do not use a typical walking pattern. Increasing AVA in our models led to substantial higher muscle activations for the majority of hip and knee muscle groups (Fig. 6), which is unlikely to be sustained over a long period. In a pilot study with a person with increased AVA we showed that the patient-specific in-toeing gait pattern can reduce muscle activations and joint contact forces to typical values [65]. This is in agreement with Alexander et al. [30] who recently showed that patients with idiopathic increased AVA and in-toing gait do not have increased hip and knee loads. In children with cerebral palsy and increased AVA, Bosmans et al. [24] showed that patient-specific gait patterns reduce hip joint loading.

Hence, it seems that, in many people with increased AVA, a pathological gait pattern is used to decrease muscular effort and potentially avoid pain due to increased joint contact forces.

Low AVA values (18° or less) in our models did not lead to higher muscle activation and joint contact forces. Hence, we assume that decreased AVA, i.e. retroversion, is not a reason for out-toeing gait. Other anatomical variations, e.g. external tibial torsion, might contribute to out-toeing gait. We only assessed the influence of femoral geometry on muscle and joint contact forces and kept the tibia the same in all models. Torsional deformities, however, often occur concurrent at the femur and tibia, which might impact gait patterns and joint loading. For example, patients with idiopathic increased AVA and internal tibial torsion walk with a typical foot progression angle [29], whereas patients with idiopathic increased AVA and typical tibial torsion values walk with in-toeing gait [30]. Future studies should, therefore, investigate how combined torsional deformities at the femur and tibia influence muscle and joint contact forces.

In our reference model the second peak of the hip joint contact force was slightly larger than the first peak, which was in agreement with many simulation studies [27,44,66,67]. In-vivo measurements of hip joint loads during walking showed on average larger or similar hip joint contact forces during the first compared to the second peak of the contact force [68,69]. In-vivo measured hip loads are highly variable between participants and some patients from the orthoload database (e.g. h2r, https://orthoload.com/) show larger hip loads during the first compared to the second peak similar to the reference simulations in our study. Different walking velocity [47,48] and bony geometries between the elderly patients with an instrumented joint replacement and young, healthy participants might explain the partially different hip joint load pattern between the simulation studies and in-vivo measurements.

Higher muscle forces, especially of the hip abductor and flexor muscles, were required to produce the necessary joint moments in models with increased AVA compared to the reference model (Fig. 8). This indicates that it might be challenging or even impossible for people with hip muscle weakness and increased AVA to walk with a typical gait pattern. A recent study [51] showed that patients with

increased AVA and in-toeing gait require less gluteus medius muscle forces compared to a healthy control group. In patient with increased AVA but normal foot progression angle, Passmore et al. [29] showed that higher gluteus medius muscle forces are needed for walking with the patient-specific femoral geometry compared to a normal, typical geometry. Several other studies [70–73] investigated the relationship between femoral geometry and muscle strength. To the best of our knowledge, no studies showed a clear relationship between the femoral geometry (combined AVA and NSA), muscle strength and gait pattern. Hence, further research is needed to determine how the patient-specific femoral geometry influences the patient's gait pattern and vice versa.

Low joint contact forces were observed in models with decreased AVA and NSA. It is important to highlight that muscle and joint contact forces only account for a small portion of all the clinically-relevant parameters. Many other factors, e.g. labral tear [74] and femoroacetabular impingement [75], could add to clinical problems in people with decreased NSA and/or reduced AVA.

We calculated muscle forces using static optimization, which might underestimate muscle co-contraction. Numerous studies showed that calibrating muscle parameters based on electromyography (EMG) signals and using an EMG-informed musculoskeletal model increases the accuracy of the simulations [41,76–80],especially in people with neurological disorders [52,81]. In our study based on 'what-if' simulations it was not possible to calibrate muscle parameters based on EMG data and therefore the absolute values of our estimated muscle and joint contact forces should be interpreted with caution. In our study, muscle forces estimated via static optimization might underestimate muscle co-contraction. Hence, our simulations showed the minimum required co-contraction for a certain AVA and NSA combination. In other words, our findings showed that you cannot avoid muscle co-contraction if you have large AVA and NSA, and walk with a normal gait pattern.

The findings of our musculoskeletal modelling study supplement previous clinical observations. A recent systematic review [8] showed that AVA 24° above reference values is associated with earlier and more severe hip osteoarthritis, whereas decreased AVA, i.e. retroversion, did show inconsistent results and

no strong correlation with the development of osteoarthritis. Our study showed that AVA of 48° largely increases hip joint contact forces particularly when associated with large (153°) NSA. On the other-hand, femoral retroversion, barely altered or even decreased hip joint contact forces. Considering that abnormal hip joint loads are a primary risk factor for the development and progression of hip osteoarthritis [82], our findings might explain why the systematic review [8] did not find any association between decreased AVA and hip osteoarthritis. A recent study [4] reported high incidence of hip (63%) and knee (58%) joint pain in children with idiopathic increased AVA. In this study [4], many children with idiopathic increased AVA walked with an in-toeing gait but some children walked with foot progression angles comparable to the control group, which made a comparison with our findings possible. We found increased hip and knee joint contact forces in models with large AVA, which suggest a link between large contact forces and the incidence of pain in these two joints. Importantly, in-toeing gait might completely change the loading situation as shown in previous studies [30,65]. A study including 111 patients with idiopathic knee osteoarthritis showed that NSA beyond 134° increases the risk of severe osteoarthritis eightfold [83]. In this study [83] the group with the most severe knee osteoarthritis had NSA between 126° and 148°. Our models with increased NSA had an NSA of 138° and 153°. Hence, our models included realistic NSA values, which are comparable to NSA in patients with idiopathic osteoarthritis. We observed increased knee joint contact forces beyond four times body weight (above 100% of the values from the reference model) in models with large NSA, which might explain the increased risk of knee osteoarthritis in patients with large NSA [83]. Early work from Tönnis and Heinecke [84] suggested that diminished AVA can cause pain and osteoarthritis. In this paper, 17% of patients with diminished AVA had high NSA (above 140°). Considering that a recent systematic review [8] did not find a correlation between diminished AVA and osteoarthritis, the increased NSA and not the reduced AVA might explain the observed pain and osteoarthritis in some of the patients from Tönnis and Heinecke [84]. However, other factors, e.g. cam/pincer deformities, additionally to the diminished AVA could have also caused pain and influenced their findings.

De-rotation osteotomies are used to correct femoral and tibial malrotations with the aim to improve the patients' gait pattern and avoid degenerative joint diseases in the long run. Indeed, femoral de-rotation osteotomies improved the gait pattern in people with idiopathic femoral AVA [85], whereas tibial de-rotation osteotomies improved the gait pattern in people with increased tibial torsion [86]. Our findings suggest that the clinical team should include the quantification and consideration of the NSA when planning de-rotation osteotomies. In people with large NSA, solely the de-rotation of the femur might not lead to a reduction in joint loads in the presence of a typical gait pattern.

A recent simulation study [28] investigated the impact of femoral AVA on patellofemoral joint loads. The authors found increased patellofemoral loads when AVA was increased from 22° to 42°, whereas a decrease in joint load was observed for the model with an AVA of 52°. This study [28] implemented a different modelling approach, i.e. muscle-driven simulations, which also altered joint kinematics and kinetics and therefore makes a direct comparison with our findings difficult. Nevertheless, the rectus femoris was found to greatly influence patellofemoral loads with altered AVA [28]. This is in agreement with the observed increases in rectus femoris forces in our models with large AVA. Combining our findings with the results from Wheatley et al. [28] further supports the hypothesis that higher rectus femoris muscle forces counterbalance the increased hip extensor muscle moments in models with large AVA (summarized in detail above), leading to increased knee and patellofemoral joint contact forces.

Our study included the following limitations. First, we only included motion capture data of one healthy participant, whereas the gait pattern, especially in people with symptomatic femoral deformities, is very heterogeneous [3,17,86]. Our musculoskeletal modelling study, however, enabled us to comprehensively quantify how muscle and joint contact forces are altered solely due to changes in femoral geometry without any confounding factors. Future studies can extend our investigation and evaluate how the patient-specific gait patterns influence muscle and joint contact forces. Second, we chose a common musculoskeletal model, i.e. gait2392, and optimization criterion, i.e. minimizing squared muscle activation, for the estimation of muscle and joint contact forces. Different musculoskeletal models and optimization approaches to solve the muscle redundancy problem might

lead to slightly different results [41,87]. Third, our musculoskeletal model included a planar knee model [88], which did not allow any out of sagittal plane movements. Furthermore, we locked the subtalar joint in our models due to an insufficient number of markers to track both, the talocrural and subtalar, joints. Considering that our simulations were based on gait data of a healthy person and subtalar joint movements are minor compared to talocrural movements [89], we believe that these limitations had a neglectable impact on our simulation results. Fourth, cartilage stress is associated with joint pain and osteoarthritis [90,91], whereas we only estimated joint contact forces. Detailed cartilage stress analyses based on finite element models were beyond the scope of our study. Future studies, however, can use our musculoskeletal simulation results (freely available on https://simtk.org/projects/bone_gait_load) as input for further biomechanical investigations to quantify cartilage stress.

We conducted 'what-if' simulation to investigate how muscle and joint contact forces would change if a person with femoral deformities would walk with a typical (normal) gait pattern. Our study design enabled us to isolate the impact of altered femoral geometry on muscle and joint contact forces and increase our fundamental understanding of femoral deformities and gait biomechanics. Our findings showed that both, the AVA and NSA, have a big impact on muscle forces and joint loads. Hence, both parameters (NSA and AVA) should be quantified and included in the clinical treatment planning. Future studies are encouraged to investigate how different patient-specific gait strategies alter joint loads in people with femoral deformities. We highly recommend to report the individual NSA and AVA of all participants in future studies to facilitate data interpretation and comparisons with other investigations.

In conclusion, we conducted a musculoskeletal modelling study and showed how altered proximal femoral geometries, i.e. AVA, NSA and combinations of AVA and NSA, influence muscle and joint contact forces during a typical gait pattern. Our simulation results suggest that large AVA lead to increases in both knee and hip contact forces. Large NSA (153°) combined with large AVA (48°) led to increases in hip joint contact forces by five times body weight. On the other hand, low NSA (108° and 93°) combined with large AVA (48°) led to two-fold increases in the second peak of the knee contact forces. Furthermore, our comprehensive analyses enabled us to put forward the hypothesis that increased hip

and knee joint contact forces in models with increased AVA and NSA are linked to changes in hip muscle moment arms and compensatory increases in hip and knee muscle forces. Our findings might help to explain clinical observations, e.g. pain, degenerative joint diseases, and why a typical gait pattern is problematic in some patients with femoral deformities.

References

1. Bobroff ED, Chambers HG, Sartoris DJ, Wyatt MP, Sutherland DH. Femoral anteversion and neck-shaft angle in children with cerebral palsy. Clin Orthop Relat Res. 1999; 194–204. Available: http://www.ncbi.nlm.nih.gov/pubmed/10416409
2. Alexander N, Studer K, Lengnick H, Payne E, Klima H, Wegener R. The impact of increased femoral antetorsion on gait deviations in healthy adolescents. J Biomech. 2019;86: 167–174. doi:10.1016/J.JBIOMECH.2019.02.005
3. Radler C, Kranzl A, Manner HM, Höglinger M, Ganger R, Grill F. Torsional profile versus gait analysis: Consistency between the anatomic torsion and the resulting gait pattern in patients with rotational malalignment of the lower extremity. Gait Posture. 2010;32: 405–410. doi:10.1016/J.GAITPOST.2010.06.019
4. Mackay J, Thomason P, Sangeux M, Passmore E, Francis K, Graham HK. The impact of symptomatic femoral neck anteversion and tibial torsion on gait, function and participation in children and adolescents. Gait Posture. 2021;86: 144–149. doi:10.1016/J.GAITPOST.2021.03.004
5. Leblebici G, Akalan E, Apti A, Kuchimov S, Kurt A, Onerge K, et al. Increased femoral anteversion-related biomechanical abnormalities: lower extremity function, falling frequencies, and fatigue. Gait Posture. 2019;70: 336–340. doi:10.1016/J.GAITPOST.2019.03.027
6. Scorcelletti M, Reeves ND, Rittweger J, Ireland A. Femoral anteversion: significance and measurement. J Anat. 2020;237: 811–826. doi:10.1111/JOA.13249
7. Pagliazzi G, De Pieri E, Kläusler M, Sangeux M, Viehweger E. Torsional deformities and overuse injuries: what does the literature tell us. EFORT Open Rev. 2022;7: 26–34. doi:10.1530/EOR-21-0092
8. Parker EA, Meyer AM, Nasir M, Willey MC, Brown TS, Westermann RW. Abnormal Femoral Anteversion Is Associated With the Development of Hip Osteoarthritis: A Systematic Review and Meta-Analysis. Arthrosc Sport Med Rehabil. 2021;3: e2047–e2058. doi:10.1016/J.ASMR.2021.07.029
9. Sangeux M. Biomechanics of the Hip During Gait. The Pediatric and Adolescent Hip. Springer International Publishing; 2019. pp. 53–71. doi:10.1007/978-3-030-12003-0_3
10. Fabry G, MacEwen GD, Shands AR. Torsion of the femur. A follow up study in normal and abnormal conditions. J Bone Jt Surg - Ser A. 1973;55: 1726–1738. doi:10.2106/00004623-197355080-00017
11. Robin J, Graham HK, Selber P, Dobson F, Smith K, Baker R. Proximal femoral geometry in cerebral palsy. Bone Joint J. 2008;90-B.
12. Mayr HO, Schmidt JP, Haasters F, Bernstein A, Schmal H, Prall WC. Anteversion Angle Measurement in Suspected Torsional Malalignment of the Femur in 3-Dimensional EOS vs Computed Tomography—A Validation Study. J Arthroplasty. 2021;36: 379–386. doi:10.1016/J.ARTH.2020.07.058
13. Paley D. Principles of Deformity Correction. Princ Deform Correct. 2002. doi:10.1007/978-3-642-59373-4
14. Uemura K, Atkins PR, Fiorentino NM, Anderson AE. Hip rotation during standing and dynamic activities and the compensatory effect of femoral anteversion: An in-vivo analysis of

asymptomatic young adults using three-dimensional computed tomography models and dual fluoroscopy. Gait Posture. 2018;61: 276–281. doi:10.1016/J.GAITPOST.2018.01.016
15. Chadayammuri V, Garabekyan T, Bedi A, Pascual-Garrido C, Rhodes J, O'Hara J, et al. Passive hip range of motion predicts femoral torsion and acetabular version. J Bone Jt Surg - Am Vol. 2016;98: 127–134. doi:10.2106/JBJS.O.00334
16. Cibulka MT, Winters K, Kampwerth T, McAfee B, Payne L, Roeckenhaus T, et al. PREDICTING FOOT PROGRESSION ANGLE DURING GAIT USING TWO CLINICAL MEASURES IN HEALTHY ADULTS, A PRELIMINARY STUDY. Int J Sports Phys Ther. 2016;11: 400. Available: /pmc/articles/PMC4886808/
17. Bruderer-Hofstetter M, Fenner V, Payne E, Zdenek K, Klima H, Wegener R. Gait deviations and compensations in pediatric patients with increased femoral torsion. J Orthop Res. 2015;33: 155–162. doi:10.1002/JOR.22746
18. Braatz F, Wolf SI, Gerber A, Klotz MC, Dreher T. Do changes in torsional magnetic resonance imaging reflect improvement in gait after femoral derotation osteotomy in patients with cerebral palsy? Int Orthop. 2013;37: 2193–2198. doi:10.1007/S00264-013-2054-7/FIGURES/2
19. Arnold AS, Komattu A V., Delp SL. Internal rotation gait: a compensatory mechanism to restore abduction capacity decreased by bone deformity? Dev Med Child Neurol. 1997;39: 40–44. doi:10.1111/J.1469-8749.1997.TB08202.X
20. Schmidt DJ, Arnold AS, Carroll NC, Delp SL. Length changes of the hamstrings and adductors resulting from derotational osteotomies of the femur. J Orthop Res. 1999;17: 279–285. doi:10.1002/JOR.1100170218
21. Arnold AS, Asakawa DJ, Delp SL. Do the hamstrings and adductors contribute to excessive internal rotation of the hip in persons with cerebral palsy? Gait Posture. 2000;11: 181–190. doi:10.1016/S0966-6362(00)00046-1
22. Arnold AS, Delp SL. Rotational moment arms of the medial hamstrings and adductors vary with femoral geometry and limb position: Implications for the treatment of internally rotated gait. J Biomech. 2001;34: 437–447. doi:10.1016/S0021-9290(00)00232-3
23. Bosmans L, Jansen K, Wesseling M, Molenaers G, Scheys L, Jonkers I. The role of altered proximal femoral geometry in impaired pelvis stability and hip control during CP gait: A simulation study. Gait Posture. 2016;44: 61–67. doi:10.1016/J.GAITPOST.2015.11.010
24. Bosmans L, Wesseling M, Desloovere K, Molenaers G, Scheys L, Jonkers I. Hip contact force in presence of aberrant bone geometry during normal and pathological gait. J Orthop Res. 2014;32: 1406–1415. doi:10.1002/jor.22698
25. Heller MO, Bergmann G, Deuretzbacher G, Claes L, Haas NP, Duda GN. Influence of femoral anteversion on proximal femoral loading: measurement and simulation in four patients. Clin Biomech. 2001;16: 644–649. doi:10.1016/S0268-0033(01)00053-5
26. Kainz H, Killen BA, Wesseling M, Perez-Boerema F, Pitto L, Garcia Aznar JM, et al. A multi-scale modelling framework combining musculoskeletal rigid-body simulations with adaptive finite element analyses, to evaluate the impact of femoral geometry on hip joint contact forces and femoral bone growth. PLoS One. 2020;15: e0235966. doi:10.1371/journal.pone.0235966
27. Modenese L, Barzan M, Carty CP. Dependency of lower limb joint reaction forces on femoral version. Gait Posture. 2021;88: 318–321. doi:10.1016/J.GAITPOST.2021.06.014
28. Wheatley BB, Chaclas NA, Seeley MA. Patellofemoral joint load and knee abduction/adduction moment are sensitive to variations in femoral version and individual muscle forces. J Orthop Res. 2022 [cited 14 Jul 2022]. doi:10.1002/JOR.25396
29. Passmore E, Graham HK, Pandy MG, Sangeux M. Hip- and patellofemoral-joint loading during gait are increased in children with idiopathic torsional deformities. Gait Posture. 2018;63: 228–235. doi:10.1016/j.gaitpost.2018.05.003
30. Alexander N, Brunner R, Cip J, Viehweger E, Pieri E De. Increased Femoral Anteversion Does Not Lead to Increased Joint Forces During Gait in a Cohort of Adolescent Patients. Front Bioeng Biotechnol. 2022;10: 1. doi:10.3389/FBIOE.2022.914990

31. Delp SL, Loan JP, Hoy MG, Zajac FE, Topp EL, Rosen JM. An interactive graphics-based model of the lower extremity to study orthopaedic surgical procedures. IEEE Trans Biomed Eng. 1990;37: 757–767. doi:10.1109/10.102791
32. Delp S, Anderson FC, Arnold AS, Loan P, Habib A, John CT, et al. OpenSim: Open-Source Software to Create and Analyze Dynamic Simulations of Movement. IEEE Trans Biomed Eng. 2007;54: 1940–1950. doi:10.1109/TBME.2007.901024
33. Veerkamp K, Kainz H, Killen BA, Jónasdóttir H, van der Krogt MM. Torsion Tool: An automated tool for personalising femoral and tibial geometries in OpenSim musculoskeletal models. J Biomech. 2021;125: 110589. doi:10.1016/J.JBIOMECH.2021.110589
34. Svoboda B, Kranzl A. A study of the reproducibility of the marker application of the Cleveland Clinic Marker Set including the Plug-In Gait Upper Body Model in clinical gait analysis. Gait Posture. 2012;36: S62–S63. doi:10.1016/J.GAITPOST.2011.10.286
35. Kainz H, Hoang H, Stockton C, Boyd RR, Lloyd DG, Carty CP. Accuracy and Reliability of Marker Based Approaches to Scale the Pelvis, Thigh and Shank Segments in Musculoskeletal Models. J Appl Biomech. 2017; 1–21. doi:10.1123/jab.2016-0282
36. Modenese L, Ceseracciu E, Reggiani M, Lloyd DG. Estimation of musculotendon parameters for scaled and subject specific musculoskeletal models using an optimization technique. J Biomech. 2016;49: 141–148. doi:10.1016/J.JBIOMECH.2015.11.006
37. Kotsifaki A, Van Rossom S, Whiteley R, Korakakis V, Bahr R, Sideris V, et al. Single leg vertical jump performance identifies knee function deficits at return to sport after ACL reconstruction in male athletes. Br J Sports Med. 2022;56: 490–498. doi:10.1136/BJSPORTS-2021-104692
38. van Veen B, Montefiori E, Modenese L, Mazzà C, Viceconti M. Muscle recruitment strategies can reduce joint loading during level walking. J Biomech. 2019;97: 109368. doi:10.1016/J.JBIOMECH.2019.109368
39. Delp S, Anderson FC, Arnold AS, Loan P, Habib A, John CT, et al. OpenSim: Open source to create and analyze dynamic simulations of movement. IEEE Trans Biomed Eng. 2007;54: 1940–1950. doi:10.1109/TBME.2007.901024
40. Steele KM, DeMers MS, Schwartz MH, Delp SL. Compressive tibiofemoral force during crouch gait. Gait Posture. 2012;35: 556–560. doi:10.1016/J.GAITPOST.2011.11.023
41. Hoang HX, Diamond LE, Lloyd DG, Pizzolato C. A calibrated EMG-informed neuromusculoskeletal model can appropriately account for muscle co-contraction in the estimation of hip joint contact forces in people with hip osteoarthritis. J Biomech. 2019;83: 134–142. doi:10.1016/J.JBIOMECH.2018.11.042
42. Schwartz MH, Rozumalski A, Trost JP. The effect of walking speed on the gait of typically developing children. J Biomech. 2008;41: 1639–1650. doi:10.1016/j.jbiomech.2008.03.015
43. Modenese L, Montefiori E, Wang A, Wesarg S, Viceconti M, Mazzà C. Investigation of the dependence of joint contact forces on musculotendon parameters using a codified workflow for image-based modelling. J Biomech. 2018;73: 108–118. doi:10.1016/j.jbiomech.2018.03.039
44. Valente G, Taddei F, Jonkers I. Influence of weak hip abductor muscles on joint contact forces during normal walking: Probabilistic modeling analysis. J Biomech. 2013;46: 2186–2193. doi:10.1016/j.jbiomech.2013.06.030
45. Saxby DJ, Modenese L, Bryant AL, Gerus P, Killen B, Fortin K, et al. Tibiofemoral contact forces during walking, running and sidestepping. Gait Posture. 2016;49: 78–85. doi:10.1016/J.GAITPOST.2016.06.014
46. Damm P, Kutzner I, Bergmann G, Rohlmann A, Schmidt H. Comparison of in vivo measured loads in knee, hip and spinal implants during level walking. J Biomech. 2017;51: 128–132. doi:10.1016/J.JBIOMECH.2016.11.060
47. Giarmatzis G, Jonkers I, Wesseling M, Van Rossom S, Verschueren S. Loading of Hip Measured by Hip Contact Forces at Different Speeds of Walking and Running. J Bone Miner Res. 2015;30: 1431–1440. doi:10.1002/jbmr.2483
48. Alexander N, Schwameder H, Baker R, Trinler U. Effect of different walking speeds on joint and

muscle force estimation using AnyBody and OpenSim. Gait Posture. 2021;90: 197–203. doi:10.1016/J.GAITPOST.2021.08.026
49. Sutherland DH. The evolution of clinical gait analysis part l: kinesiological EMG. Gait Posture. 2001;14: 61–70. doi:10.1016/S0966-6362(01)00100-X
50. Agostini V, Nascimbeni A, Gaffuri A, Imazio P, Benedetti MG, Knaflitz M. Normative EMG activation patterns of school-age children during gait. Gait Posture. 2010;32: 285–289. doi:10.1016/J.GAITPOST.2010.06.024
51. De Pieri E, Cip J, Brunner R, Weidensteiner C, Alexander N. The functional role of hip muscles during gait in patients with increased femoral anteversion. Gait Posture. 2023;100: 179–187. doi:10.1016/J.GAITPOST.2022.12.014
52. Kainz H, Hoang H, Pitto L, Wesseling M, Van Rossom S, Van Campenhout A, et al. Selective dorsal rhizotomy improves muscle forces during walking in children with spastic cerebral palsy. Clin Biomech (Bristol, Avon). 2019;65: 26–33. doi:10.1016/j.clinbiomech.2019.03.014
53. Kainz H, Hajek M, Modenese L, Saxby DJ, Lloyd DG, Carty CP. Reliability of functional and predictive methods to estimate the hip joint centre in human motion analysis in healthy adults. Gait Posture. 2017;53: 179–184. doi:10.1016/j.gaitpost.2017.01.023
54. Wu G, Siegler S, Allard P, Kirtley C, Leardini A, Rosenbaum D, et al. ISB recommendation on definitions of joint coordinate system of various joints for the reporting of human joint motion—part I: ankle, hip, and spine. J Biomech. 2002;35: 543–548. doi:10.1016/S0021-9290(01)00222-6
55. Kainz H, Modenese L, Lloyd DG, Maine S, Walsh HPJ, Carty CP. Joint kinematic calculation based on clinical direct kinematic versus inverse kinematic gait models. J Biomech. 2016;49: 1658–1669. doi:10.1016/j.jbiomech.2016.03.052
56. Koller W, Baca A, Kainz H. Impact of scaling errors of the thigh and shank segments on musculoskeletal simulation results. Gait Posture. 2021;87: 65–74. doi:10.1016/j.gaitpost.2021.02.016
57. Holder J, Trinler U, Meurer A, Stief F. A Systematic Review of the Associations Between Inverse Dynamics and Musculoskeletal Modeling to Investigate Joint Loading in a Clinical Environment. Front Bioeng Biotechnol. 2020;8: 1382. doi:10.3389/FBIOE.2020.603907/BIBTEX
58. Richards RE, Andersen MS, Harlaar J, van den Noort JC. Relationship between knee joint contact forces and external knee joint moments in patients with medial knee osteoarthritis: effects of gait modifications. Osteoarthr Cartil. 2018;26: 1203–1214. doi:10.1016/J.JOCA.2018.04.011
59. Szuper K, Schlégl ÁT, Leidecker E, Vermes C, Somoskeöy S, Than P. Three-dimensional quantitative analysis of the proximal femur and the pelvis in children and adolescents using an upright biplanar slot-scanning X-ray system. Pediatr Radiol. 2015;45: 411–421. doi:10.1007/s00247-014-3146-2
60. Pierrepont JW, Marel E, Baré J V., Walter LR, Stambouzou CZ, Solomon MI, et al. Variation in femoral anteversion in patients requiring total hip replacement. Hip Int. 2020;30: 281–287. doi:10.1177/1120700019848088
61. Terjesen T, Benum P, Anda S, Svenningsen S. Increased Femoral Anteversion and Osteoarthritis of the Hip Joint. https://doi.org/103109/17453678208992260. 2009;53: 571–575. doi:10.3109/17453678208992260
62. Kumar D, Manal KT, Rudolph KS. Knee joint loading during gait in healthy controls and individuals with knee osteoarthritis. Osteoarthr Cartil. 2013;21: 298–305. doi:10.1016/J.JOCA.2012.11.008
63. De Pieri E, Friesenbichler B, List R, Monn S, Casartelli NC, Leunig M, et al. Subject-specific modelling of femoral torsion influences the prediction of hip loading during gait in asymptomatic adults. Front Bioeng Biotechnol. 2021;0: 551. doi:10.3389/FBIOE.2021.679360
64. Koller W, Baca A, Kainz H. Asymmetric gait pattern versus femoral morphology – What is the main reason for asymmetric hip joint loading? Gait Posture. 2021;90: 125–126.

doi:10.1016/J.GAITPOST.2021.09.065
65. Kainz H, Kranzl A. In-toeing gait requires less muscular effort and reduces lower limb joint loads in people with internal torsional deformities. Gait Posture. 2022;97: S391–S392. doi:10.1016/J.GAITPOST.2022.07.239
66. Kainz H, Killen BA, Wesseling M, Perez-Boerema F, Pitto L, Manuel J, et al. A multi-scale modelling framework combining musculoskeletal rigid-body simulations with adaptive finite element analyses, to evaluate the impact of femoral geometry on hip joint contact forces and femoral bone growth. 2020. doi:10.1371/journal.pone.0235966
67. Varady PA, Glitsch U, Augat P. Loads in the hip joint during physically demanding occupational tasks: A motion analysis study. J Biomech. 2015;48: 3227–3233. doi:10.1016/J.JBIOMECH.2015.06.034
68. Haffer H, Popovic S, Martin F, Hardt S, Winkler T, Damm P. In vivo loading on the hip joint in patients with total hip replacement performing gymnastics and aerobics exercises. Sci Reports |. 123AD;11: 13395. doi:10.1038/s41598-021-92788-7
69. Palmowski Y, Popović S, Kosack D, Damm P. Analysis of hip joint loading during walking with different shoe types using instrumented total hip prostheses. Sci Reports 2021 111. 2021;11: 1–9. doi:10.1038/s41598-021-89611-8
70. V A, F S, A I, A M-L, S H, V K-I. Relative abductor strength after varus and valgus osteotomy of the proximal femur. Acta Chir Orthop Traumatol Cech. 1995;62: 354–356. Available: https://europepmc.org/article/med/20470528
71. Hogg JA, Ackerman T, Nguyen AD, Ross SE, Schmitz RJ, Vanrenterghem J, et al. The Effects of Gluteal Strength and Activation on the Relationship Between Femoral Alignment and Functional Valgus Collapse During a Single-Leg Landing. J Sport Rehabil. 2021;30: 942–951. doi:10.1123/JSR.2019-0528
72. Marostica AD, Pizzolatti ALA, Adam GP, Codonho D, Canella RP, Ganev GG. Is Femoral Version Associated with Changes in Hip Muscle Strength in Females with Symptomatic Femoroacetabular Impingement? Rev Bras Ortop. 2019;54: 422. doi:10.1055/S-0039-1694019
73. Liu J, Lewton KL, Colletti PM, Powers CM. Hip Adduction during Running: Influence of Sex, Hip Abductor Strength and Activation, and Pelvis and Femur Morphology. Med Sci Sports Exerc. 2021;53: 2346–2353. doi:10.1249/MSS.0000000000002721
74. Wenger DE, Kendell KR, Miner MR, Trousdale RT. Acetabular labral tears rarely occur in the absence of bony abnormalities. Clin Orthop Relat Res. 2004;426: 145–150. doi:10.1097/01.BLO.0000136903.01368.20
75. Lerch TD, Antioco T, Boschung A, Meier MK, Schmaranzer F, Novais EN, et al. Hip Impingement Location in Maximal Hip Flexion in Patients With Femoroacetabular Impingement With and Without Femoral Retroversion. https://doi.org/101177/03635465221110887. 2022;50: 2989–2997. doi:10.1177/03635465221110887
76. Pizzolato C, Lloyd DG, Sartori M, Ceseracciu E, Besier TF, Fregly BJ, et al. CEINMS: A toolbox to investigate the influence of different neural control solutions on the prediction of muscle excitation and joint moments during dynamic motor tasks. J Biomech. 2015;48: 3929–3936. doi:10.1016/j.jbiomech.2015.09.021
77. Falisse A, Van Rossom S, Jonkers I, De Groote F. EMG-Driven Optimal Estimation of Subject-Specific Hill Model Muscle-Tendon Parameters of the Knee Joint Actuators. IEEE Trans Biomed Eng. 2017;64: 2253–2262. doi:10.1109/TBME.2016.2630009
78. Kian A, Pizzolato C, Halaki M, Ginn K, Lloyd D, Reed D, et al. Static optimization underestimates antagonist muscle activity at the glenohumeral joint: A musculoskeletal modeling study. J Biomech. 2019;97: 109348. doi:10.1016/J.JBIOMECH.2019.109348
79. Trepczynski A, Kutzner I, Schwachmeyer V, Heller MO, Pfitzner T, Duda GN. Impact of antagonistic muscle co-contraction on in vivo knee contact forces. J Neuroeng Rehabil. 2018;15: 1–10. doi:10.1186/S12984-018-0434-3/FIGURES/3
80. Bennett KJ, Pizzolato C, Martelli S, Bahl JS, Sivakumar A, Atkins GJ, et al. EMG-Informed

Neuromusculoskeletal Models Accurately Predict Knee Loading Measured Using Instrumented Implants. IEEE Trans Biomed Eng. 2022;69: 2268–2275. doi:10.1109/TBME.2022.3141067
81. Veerkamp K, Schallig W, Harlaar J, Pizzolato C, Carty CP, Lloyd DG, et al. The effects of electromyography-assisted modelling in estimating musculotendon forces during gait in children with cerebral palsy. J Biomech. 2019 [cited 28 May 2019]. doi:10.1016/J.JBIOMECH.2019.05.026
82. Felson DT. Osteoarthritis as a disease of mechanics. Osteoarthr Cartil. 2013;21: 10–15. doi:10.1016/j.joca.2012.09.012
83. Coskun Benlidayi I, Guzel R, Basaran S, Aksungur EH, Seydaoglu G. Is coxa valga a predictor for the severity of knee osteoarthritis? A cross-sectional study. Surg Radiol Anat. 2015;37: 369–376. doi:10.1007/S00276-014-1359-6/TABLES/2
84. Tönnis D, Heinecke A. Diminished femoral antetorsion syndrome: a cause of pain and osteoarthritis. J Pediatr Orthop. 1991;11: 419–431. doi:10.1097/01241398-199107000-00001
85. MacWilliams BA, McMulkin ML, Davis RB, Westberry DE, Baird GO, Stevens PM. Biomechanical changes associated with femoral derotational osteotomy. Gait Posture. 2016;49: 202–206. doi:10.1016/J.GAITPOST.2016.07.002
86. Alexander N, Wegener R, Lengnick H, Payne E, Klima H, Cip J, et al. Compensatory gait deviations in patients with increased outward tibial torsion pre and post tibial derotation osteotomy. Gait Posture. 2020;77: 43–51. doi:10.1016/J.GAITPOST.2020.01.011
87. Wesseling M, Derikx LC, Groote F de, Bartels W, Meyer C, Verdonschot N, et al. Muscle optimization techniques impact the magnitude of calculated hip joint contact forces. J Orthop Res. 2015;33: 430–438. doi:10.1002/JOR.22769
88. Yamaguchi GT, Zajac FE. A planar model of the knee joint to characterize the knee extensor mechanism. J Biomech. 1989;22: 1–10. doi:10.1016/0021-9290(89)90179-6
89. Schallig W, van den Noort JC, McCahill J, Stebbins J, Leardini A, Maas M, et al. Comparing the kinematic output of the Oxford and Rizzoli Foot Models during normal gait and voluntary pathological gait in healthy adults. Gait Posture. 2020;82: 126–132. doi:10.1016/J.GAITPOST.2020.08.126
90. Buckwalter JA, Anderson DD, Brown TD, Tochigi Y, Martin JA. The Roles of Mechanical Stresses in the Pathogenesis of Osteoarthritis: Implications for Treatment of Joint Injuries. Cartilage. 2013;4: 286. doi:10.1177/1947603513495889
91. DeFrate LE, Kim-Wang SY, Englander ZA, McNulty AL. Osteoarthritis year in review 2018: mechanics. Osteoarthr Cartil. 2019;27: 392–400. doi:10.1016/J.JOCA.2018.12.011

Acknowledgements

We would like to thank Dr. Basilio Goncalves for his constructive feedback on the first draft of our manuscript.

Figure legends

Figure 1. Joint kinematic (top two rows) and kinetics (button two rows). The obtained waveforms from all our models were identical. Red, dashed vertical lines indicate the end of the stance phase.

Figure 2. Resultant hip, knee and ankle joint contact forces obtained with the different models. The geometry (NSA and AVA) had a big impact on hip and knee joint contact forces. Large AVA combined with large NSA led to joint contact forces up to 12 times body weight. It is unlikely that a person would walk with such high joint contact forces. People with torsional deformities are able to decrease joint contact forces with a pathological gait pattern, e.g. in-toeing gait, which should be kept in mind when interpreting our findings.

Figure 3. Scatterplots showing the relationship between anteversion angle (AVA) and mean joint contact forces during the stance phase, and neck-shaft angle (NSA) and mean joint contact forces during the stance phase. Filled circles indicate significant predictors ($p<0.001$) from the multiple regression analysis. BW=body weight. Straight lines are the regression lines obtained from the multiple regression analysis.

Figure 4. Agonist and antagonist muscle moments obtained with model with different femoral geometry. Both, altered AVA and NSA, had an influence on the obtained muscle moments. In the NSA 153° AVA 48° model, the increased first peak of the knee flexion moment was mainly caused by an increased muscle force and moment of the rectus femoris muscle.

Figure 5. Scatterplots showing the relationship between the femoral geometry (AVA and NSA) and mean co-contraction indices (CCI) and mean muscle moments during the stance phase of the gait cycle. Filled circles indicate significant predictors ($p<0.05$) from the multiple regression analysis. Straight lines are the regression lines obtained from the multiple regression analysis. CCI values of 0 indicate full co-contraction. CCI values of 1 and -1 indicate only antagonist activation and only agonist activation, respectively.

Figure 6. Scatterplots showing the relationship between the femoral geometry (AVA and NSA) and mean muscle activations during the stance phase of the gait cycle. Filled circles indicate significant predictors ($p<0.05$) from the multiple regression analysis. Straight lines are the regression lines obtained from the multiple regression analysis.

Figure 7. Average moment arm during the stance phase of gait obtained from agonist and antagonist muscle groups. Moment arms of agonist and antagonist are visualized in the same subplots as bar plots with either positive or negative values, respectively. Muscles were grouped based on their average moment arm in each anatomical plane during the stance phase of the gait cycle.

Figure 8. Average muscle forces during the stance phase of gait obtained from agonist and antagonist muscle groups. Muscle forces of agonist and antagonist are visualized in the same subplots as bar plots with either positive or negative values, respectively. Muscles were grouped based on their average moment arm in each anatomical plane during the stance phase of the gait cycle (same as in figure 7).

Figure 1

Click here to access/download;Figure;Figure_1.tif

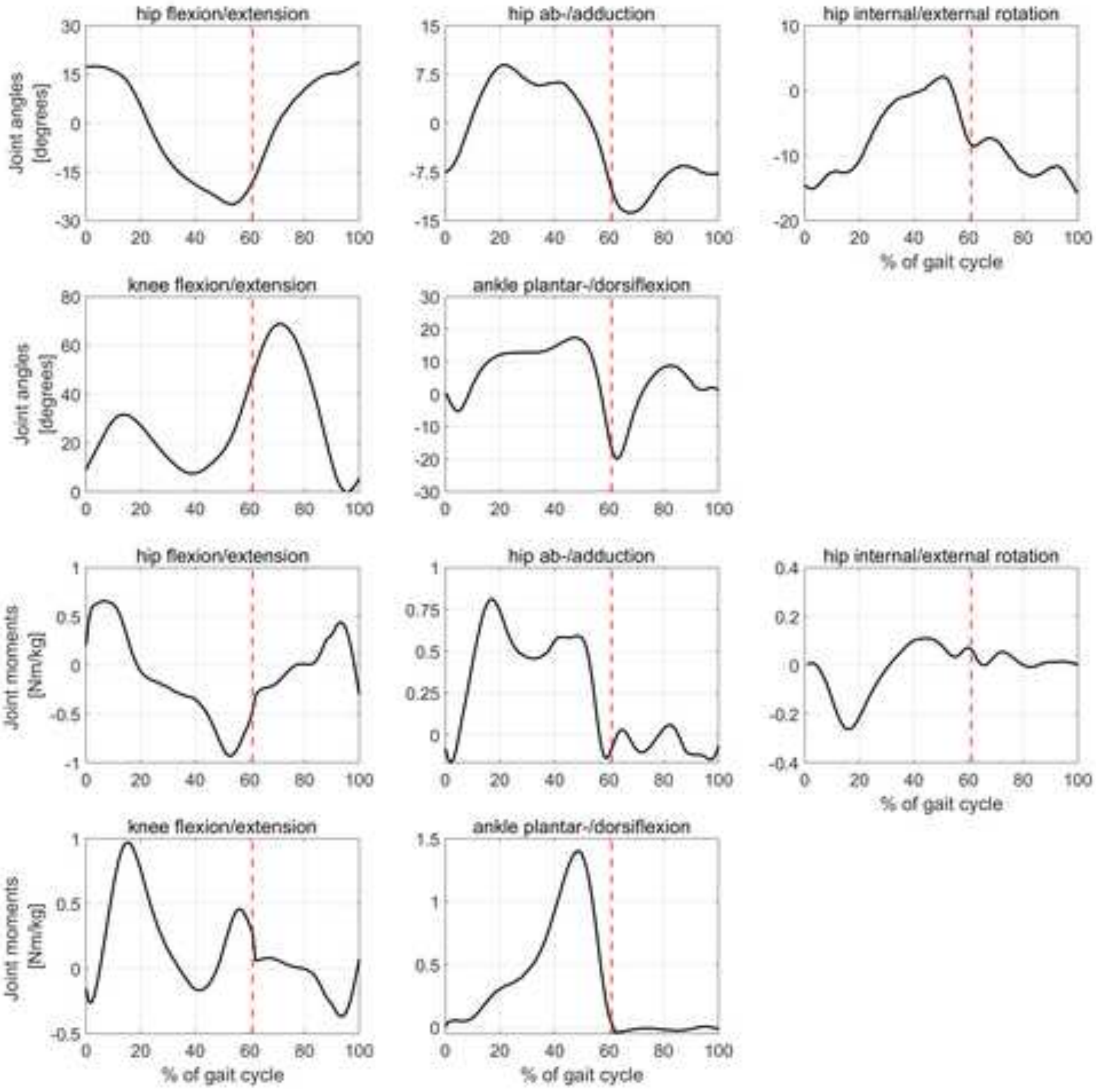

Figure 2

Click here to access/download;Figure;Figure_2.tif

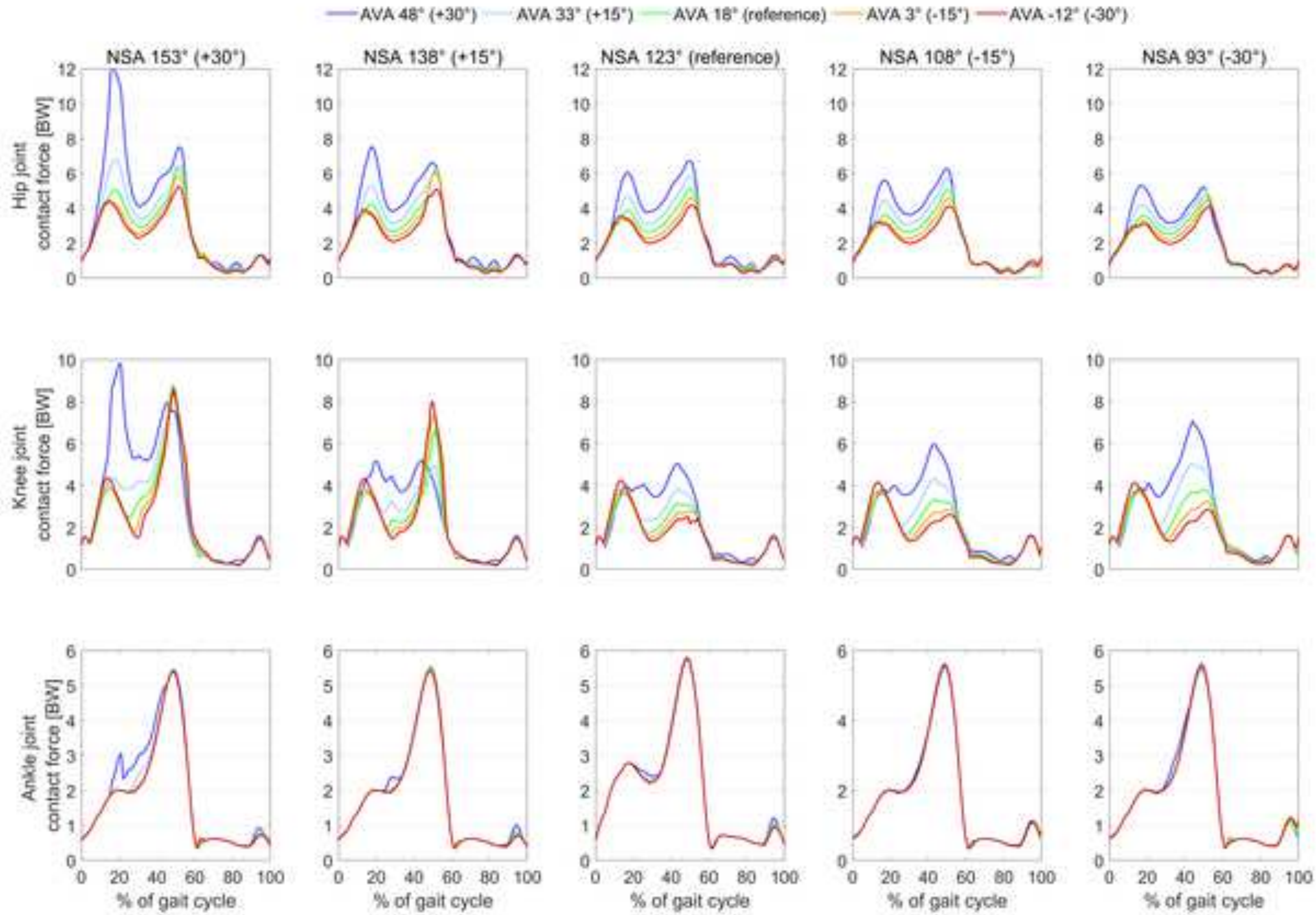

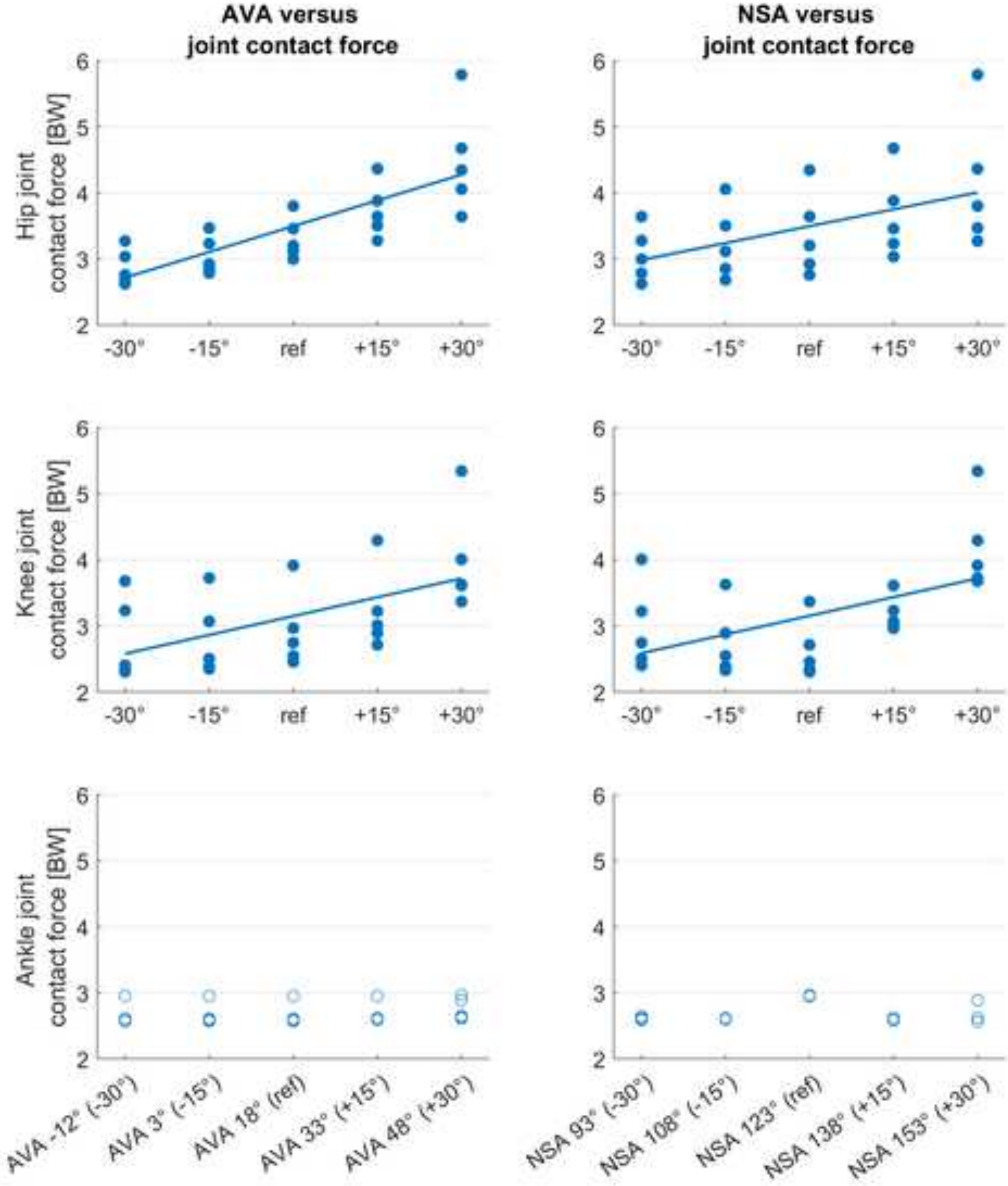
AVA versus
joint contact force
NSA versus
joint contact force
Hip joint
contact force [BW]
Knee joint
contact force [BW]
Ankle joint
contact force [BW]
2
3
4
5
6
-30°
-15°
ref
+15°
+30°
AVA -12° (-30°)
AVA 3° (-15°)
AVA 18° (ref)
AVA 33° (+15°)
AVA 48° (+30°)
NSA 93° (-30°)
NSA 108° (-15°)
NSA 123° (ref)
NSA 138° (+15°)
NSA 153° (+30°)

Figure 4

Click here to access/download;Figure;Figure_4.tif

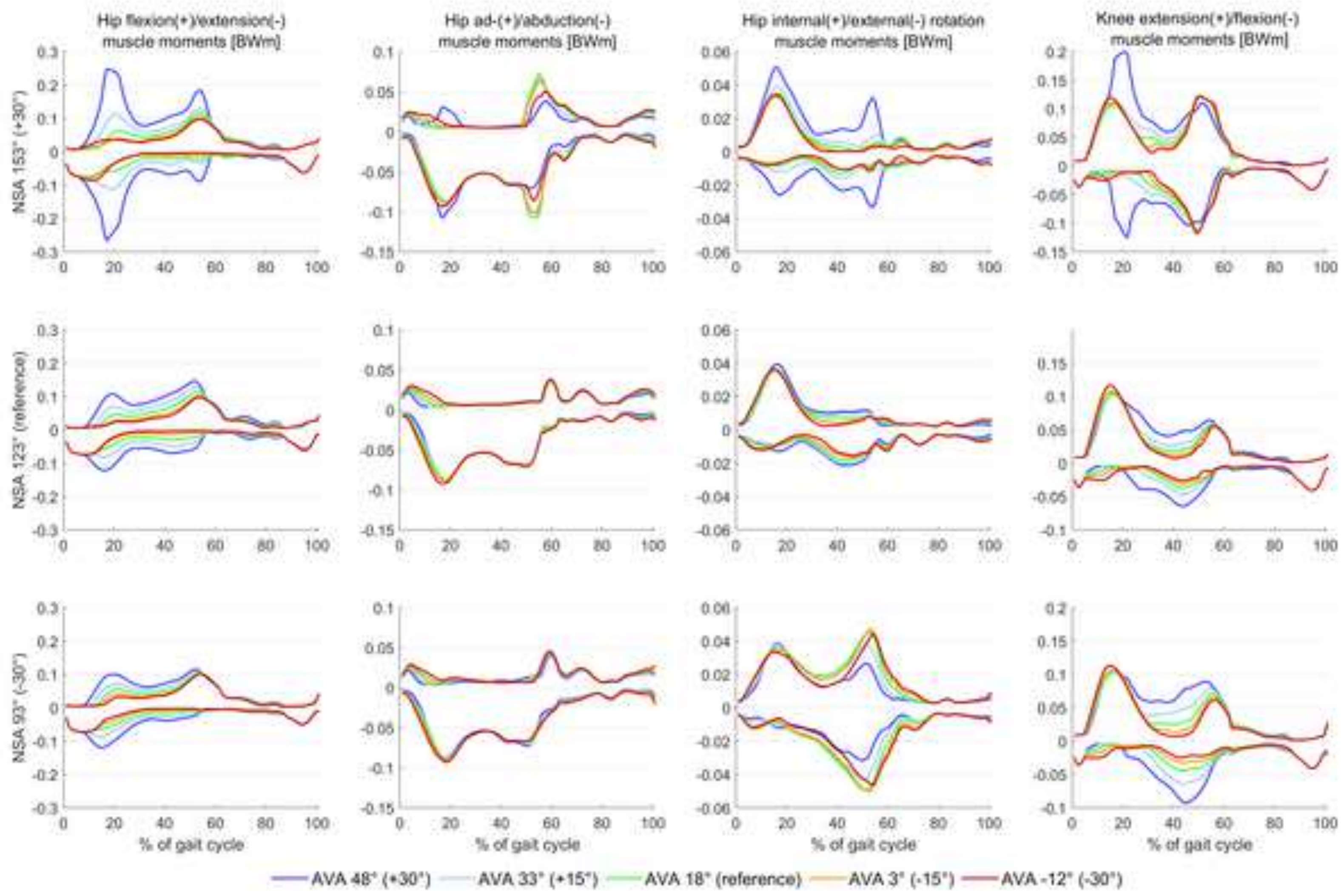

Figure 5

Click here to access/download;Figure;Figure_5.tif

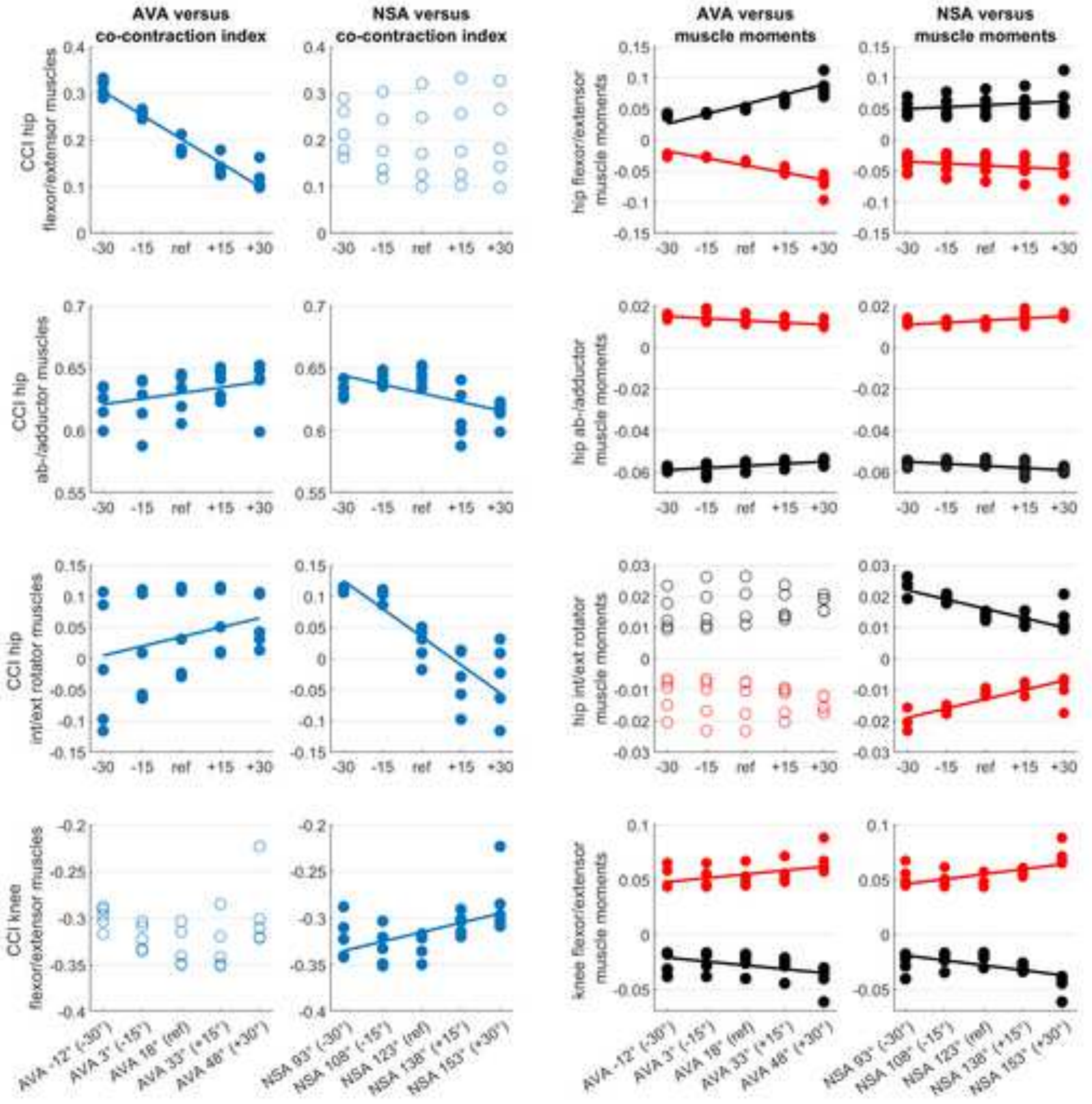

Figure 6 Click here to access/download;Figure;Figure_6.tif

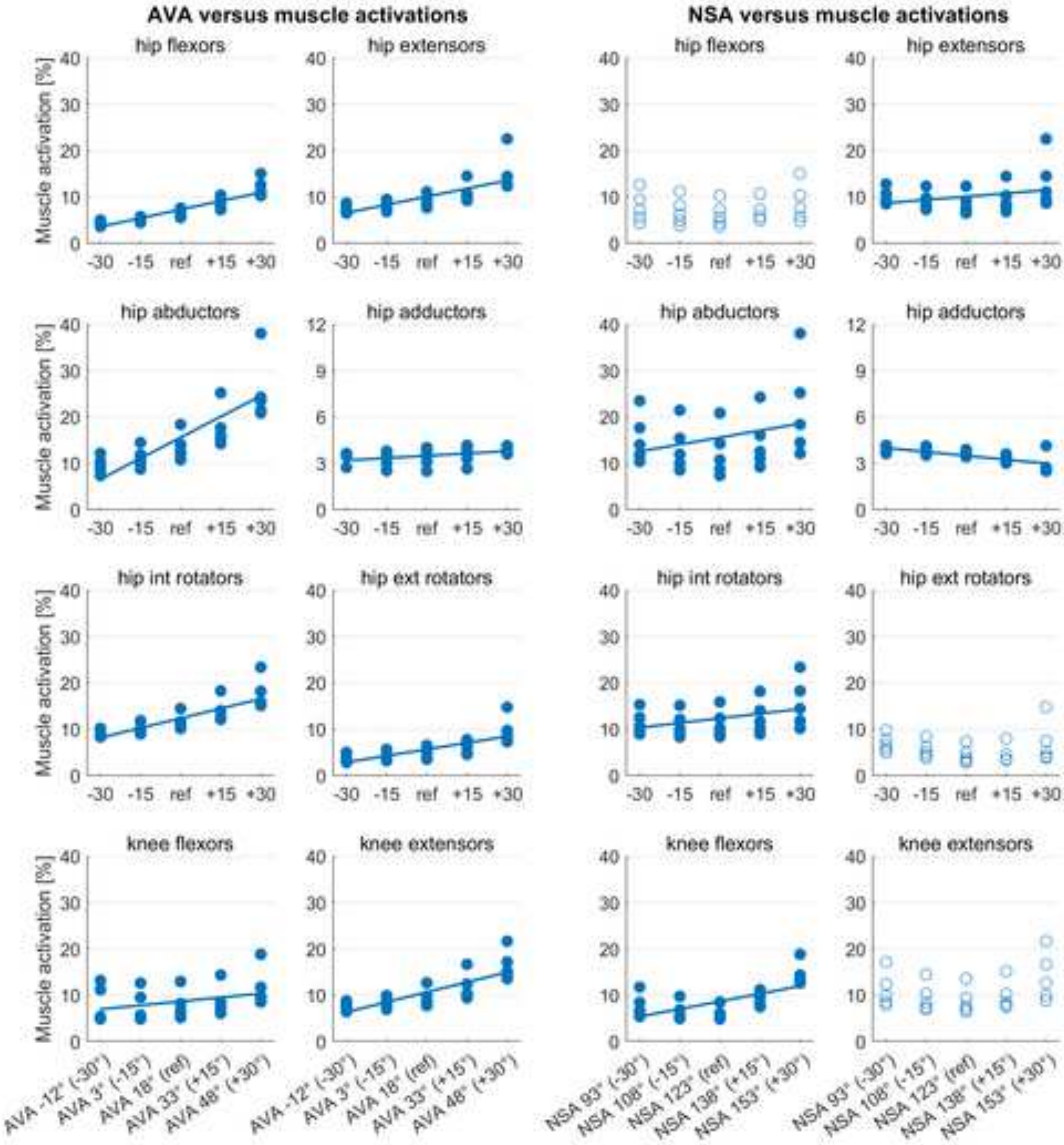

Figure 7

Click here to access/download;Figure;Figure_7.tif

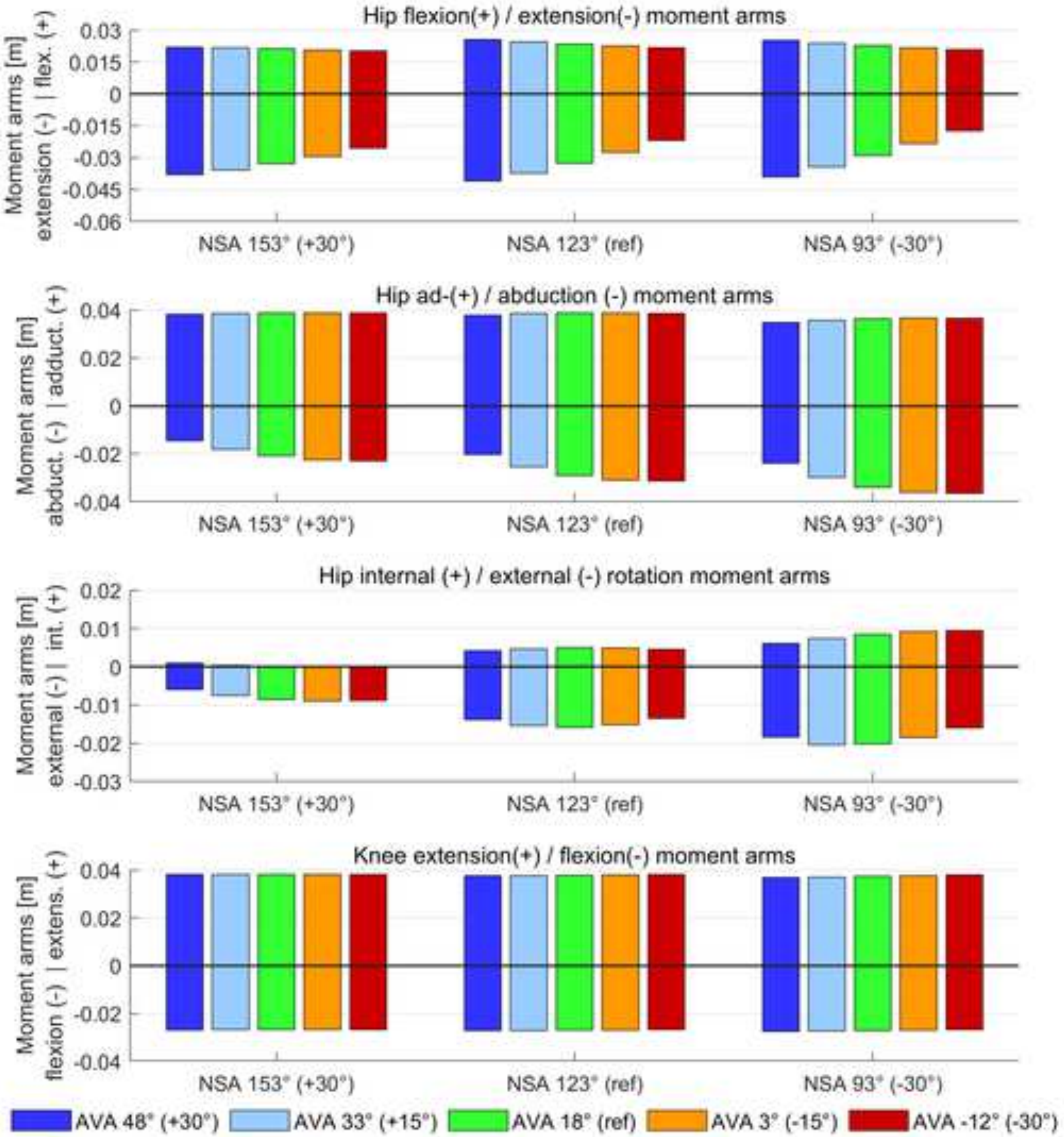

Figure 8

Click here to access/download;Figure;Figure_8.tif

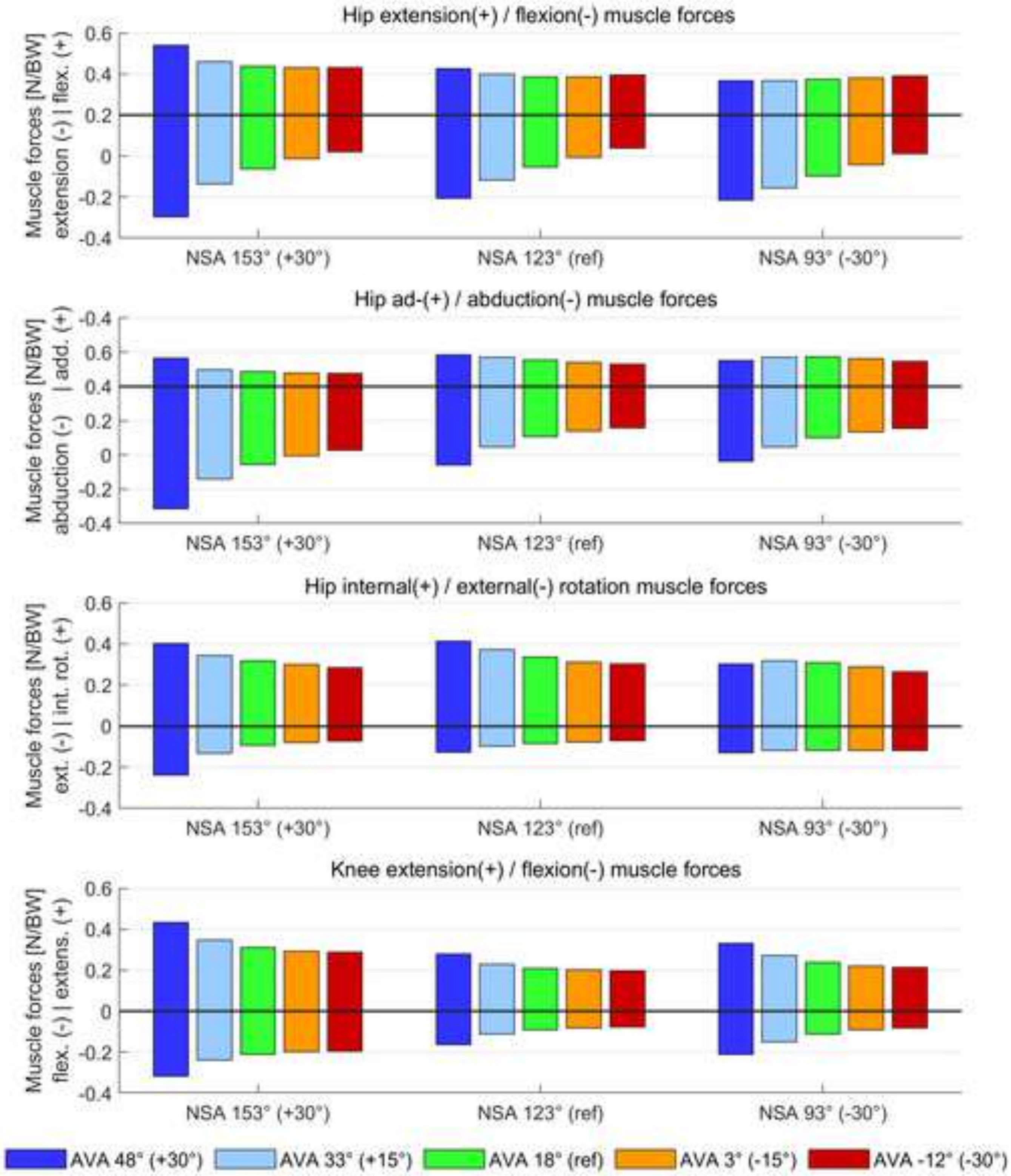

Click here to access/download
Supporting Information
SupplementaryMaterial.docx
