# Supplementary material for: Influence of femoral anteversion angle and neck-shaft angle on muscle forces and joint loading during walking
Source: PLoS One. 2023 Oct 12;18(10):e0291458. doi: 10.1371/journal.pone.0291458 (PMC10569567; doi:10.1371/journal.pone.0291458)
Supplement: Attachment — Submitted filename: RESPONSE TO REVIEWERS.docx [file pone.0291458.s005.docx]

**RESPONSE TO REVIEWERS’ COMMENTS**

Journal: PLOS ONE

Manuscript Number: PONE-D-23-11896

Title: Influence of femoral anteversion angle and neck-shaft angle on muscle forces and joint loading during walking

We thank the Editor and Reviewer for reviewing our manuscript and are thankful for the final feedback provided by the Reviewer. Please find below the Reviewer’s comment and Authors’ responses. All changes to the manuscript are shown in red font in the text below.

**Reviewer #1**

**Comment reviewer #1:**

Thank you for addressing all of my questions. Thank you for shortening the manuscript. If the authors are willing, I do believe it would be useful to add the paragraph they proposed in their response about muscle activations.

**Authors’ response:**

As suggested by the reviewer, we added the following paragraph to our discussion section:

‘Muscle activations of the reference model led to unrealistic high values. Hence, we had to multiply the maximum isometric muscle forces of the reference model by a factor of two to generate realistic muscle activation waveforms. The unrealistic high muscle activations were probably caused by a combination of the large body weight (73.1 kg) and fast walking velocity (1.41 m/s) of our participant [85]. Furthermore, we used the gait2392 model, which is known to include relatively low isometric muscle forces, which might not be representative for younger people. Newer models, e.g. Rajagopal model [86], include more realistic muscle properties (e.g. 6194 N instead of 3549 N for maximum isometric forces of the soleus). We, however, could not use the Rajagopal model for our study because the Torsion Tool [28] enables the modification of the AVA and NSA only for the gait2392 model. We plan to update the Torsion Tool in the near future to enable the personalization of the femoral and tibial geometries in all OpenSim models.’
